# CircFUNDC1 interacts with CDK9 to promote mitophagy in nucleus pulposus cells under oxidative stress and ameliorates intervertebral disc degeneration

**DOI:** 10.1038/s41419-025-07425-2

**Published:** 2025-02-13

**Authors:** Tianyuan Gu, Yong He, Jianan Zhou, Xiaoming Qiu, Wentao Yang, Qiong Zhu, Yi Liang, Yang Zheng, Jasper H. N. Yik, Dominik R. Haudenschild, Shunwu Fan, Chao Liu, Wenli Shi, Shasha Yao, Weiyu Ni, Ziang Hu

**Affiliations:** 1 Department of Orthopaedic Surgery, Sir Run Run Shaw Hospital, Zhejiang University School of Medicine, Hangzhou, Zhejiang China; 2 Key Laboratory of Musculoskeletal System Degeneration and Regeneration Translational Research of Zhejiang Province, Hangzhou, Zhejiang China; 3 Department of Radiology, Nanjing Drum Tower Hospital Clinical College of Nanjing Medical University, Nanjing, Jiangsu China; 4 Houston Methodist Research Institute, Department of Translational Orthopedic Research, Houston, TX US; 5 Orthopedics and Sports Medicine, Houston Methodist Hospital, Houston, TX US

**Keywords:** Mitophagy, Mechanisms of disease

## Abstract

Intervertebral disc degeneration (IVDD) is a leading cause of low back pain, with limited effective treatments due to an incomplete understanding of disease mechanisms. In this study, we report that circFUNDC1, a nuclear circular RNA, is markedly downregulated in nucleus pulposus cells (NPCs) from patients with end-stage IVDD. CircFUNDC1 is derived from the gene encoding the FUN14 domain-containing 1 (FUNDC1) protein, which is essential for mitophagy and cell survival. Functional analyses reveal that circFUNDC1 plays a crucial role in maintaining extracellular matrix homeostasis by enhancing the expression of anabolic factors in NPCs. Additionally, we identified the transcriptional regulator cyclin-dependent kinase 9 (CDK9) as a novel binding partner for circFUNDC1. Binding with circFUNDC1 recruits CDK9 via complementary nucleotides to the FUNDC1 promoter to stimulate the production of full-length FUNDC1 mRNAs and proteins, forming a positive feedback loop. Overexpression of circFUNDC1 protects NPCs from oxidative stress by promoting mitophagy, reducing reactive oxygen species levels, and inhibiting cellular senescence. Moreover, circFUNDC1 overexpression delays the onset of IVDD in an ex-vivo culture model. This study is the first to demonstrate that circFUNDC1 is vital for protecting NPCs from oxidative stress, suggesting circFUNDC1 as a potential therapeutic target for IVDD.

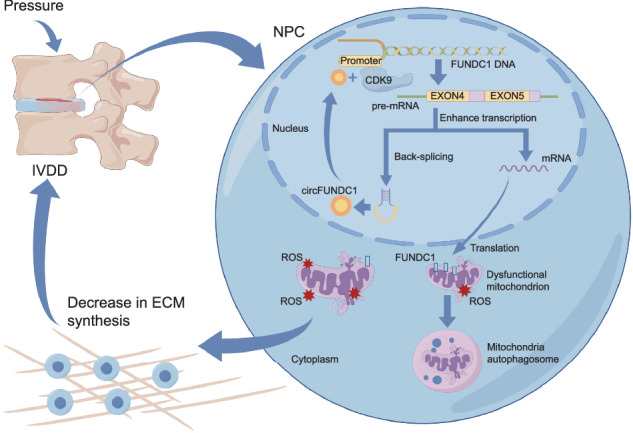

## Introduction

Low back pain has emerged as a major global health issue and a significant socioeconomic burden that will likely worsen with the aging population [1, 2]. Numerous studies indicate that low back pain is closely associated with intervertebral disc degeneration (IVDD) [3], which involves the loss of resident cells, or nucleus pulposus cells (NPCs), and extracellular matrix (ECM) [4, 5]. The ECM of intervertebral disc is primarily composed of collagen fibrils with interspersing proteoglycans (PGs), such as aggrecan. This ECM is mainly produced and maintained by NPCs [6, 7] and anabolic factors like SOX9, aggrecan, and collagen II, play a crucial role in this process [8]. Previous studies have found that senescence, a state of permanent cell cycle arrest mainly regulated by the p16-pRb and p21-p53 pathways, is closely associated with the initiation and progression of IVDD [9, 10]. Among various factors leading to cell senescence, reactive oxygen species (ROS) play a major role in mitochondrial dysfunction and deteriorating ECM [11]. Traditional conservative and surgical treatments have been widely performed to alleviate low back pain, but they often cause various adverse effects. It is critical to develop new and safer treatments that address not only the clinical symptoms but also the etiology of IVDD [12].

Biologics are well known for their safety and lack of tolerance development, making them ideal candidates in many situations. It is reasonable to infer that biologics targeting NPCs and ECM production offer a promising therapeutic approach for IVDD and other degenerative disease [13–15]. Notably, recent studies have identified long noncoding RNAs (lncRNAs) as key players in degeneration process [16–18]. Among various lncRNAs, circular RNAs (circRNAs) stand out due to their prevalence in different cell types and their unique closed-loop structure formed by head-to-tail splicing of linear RNA. This structure gives circRNAs remarkable stability by resisting exonuclease degradation, and thus making them ideal biologics [19].

There are two types of circRNAs: those located in the cytoplasm and those in the nucleus, each with unique functions. Cytoplasmic circRNAs often act as competing endogenous RNAs (ceRNAs) against microRNAs (miRNAs) and have been extensively studied in the context of IVDD [20, 21]. In contrast, nuclear circRNAs are believed to regulate gene expression directly by binding to various nuclear proteins [22–24]. Our previous work mainly focused on cytoplasmic circRNAs [25, 26]. During the course of our research, we noted that nuclear circRNAs have potential in regulating cellular transcription due to their presence at the transcription initiation stage. However, these nuclear circRNAs have been rarely studied in the context of IVDD.

From the sequencing data of a previous study on nuclear circRNAs that may regulate transcription by binding to RNA Polymerase II [27], we identified a nuclear circRNA, named hsa_circFUNDC1 (aka hsa_circ_0007290), that is significantly downregulated in NPCs isolated from patients with end-stage IVDD. The nuclear hsa_circFUNDC1, hereafter referred to as circFUNDC1, is derived from the gene encoding the FUN14 domain containing 1 (FUNDC1) protein, which is crucial for mitochondrial autophagy, or mitophagy [28, 29]. We hypothesized that the disease-associated circFUNDC1 is involved in the pathogenesis of IVDD and investigated the functions of circFUNDC1 in NPCs and in the maintenance of intervertebral disks.

## Materials and methods

### Isolation and culturing of human NPCs and nucleus pulposus

Human nucleus pulposus tissues were obtained from discarded samples of 150 patients (88 males, 62 females) who underwent intervertebral disc excision surgery, following IRB approval 20220103-51 from Ethics Committee of Sir Run Run Shaw Hospital, Affiliated with Zhejiang University School of Medicine. Radiographic diagnoses of IVDD severity were confirmed with histological analysis. Specimens with Grade I (mild) and Grade V (severe) diseases were utilized in this study. The tissues were used for RNA extraction, ex-vivo culture, and NPC isolation. To isolate NPCs, the nucleus pulposus was cut into small pieces and treated with 10 ml DMEM media (Gibco-Thermo Fisher Scientific, Waltham, MA, USA) with 0.1% collagenase type II (Biosharp, Hefei, China) for 4 to 6 h at 37 °C. Liberated cells were filtered through a 40 µm cell strainer, and the filtrate was centrifuged for 5 min at 1200 rpm. The supernatant was aspirated, and the cell pellets were washed with sterile phosphate-buffered saline (PBS), then cultured in Dulbecco’s modified Eagle’s medium (DMEM) with 10% fetal bovine serum (FBS) (Thermo Fisher Scientific, Waltham, MA, USA) at 37 °C in 5% CO2. The isolated NPCs were used within three passages for all experiments.

### Isolation and culturing of rat NPCs

The rat NPCs were separated from the lumbar disks of Sprague Dawley rats (male, 250 g, and 8 weeks old) using a dissecting microscope and finely diced into small pieces. The samples were digested with 0.1% collagenase type II (Biosharp, Hefei, China) for 4 h at 37 °C. After filtration through a 40 μm pore size mesh, rat NPCs were cultured in DMEM and Ham’s F-12 medium (DMEM/F12) (Gibco-Thermo Fisher Scientific, Waltham, MA, USA) supplemented with 10% FBS (Gibco, Gaithersburg, MD, United States) in a humidified atmosphere containing 5% CO_2_ at 37 °C.

### Cell lines and cultures

HEK-293T cells were obtained from the American Type Culture Collection (Manassas, VA, USA) and maintained in DMEM (Gibco-Thermo Fisher Scientific, Waltham, MA, USA) containing 10% FBS at 37 °C in 5% CO_2_.

### Ex-vivo cultures

Human nucleus pulposus tissues were obtained from 30 patients with Grade I or II diseases and placed on 6-well plates floating on culture medium, which was a mixture of DMEM (Gibco-Thermo Fisher Scientific, Waltham, MA, USA) supplemented with 10% fetal bovine serum (FBS) (v/v), 20U/ml Penicillin-Streptomycin (Gibco-Thermo Fisher Scientific, Waltham, MA, USA) and 4 μg/ml tetracycline hydrochloride solution (Sangon, Shanghai, China). 6-well plates were maintained in a tissue culture incubator at 37 °C with 5% CO2. The culture media was replaced every 2 days. To overexpress circFUNDC1 in Human nucleus pulposus tissues, adenovirus (pAdeasy-EF1- hsa_circ_0007290-CMV-EGFP-N) was used (100MOI). After the two-day treatment, the experimental group was treated with H_2_O_2_ (200 μM). The samples were collected after another two-day treatment and collected for IHC.

### A rat model of IVDD

All animal protocols were performed in accordance with standard ethical guidelines as approved by the Ethics Committee of Institutional Animal Care and Use Committee (IACUC, ZJCLA-20010647). A total of 24 male Sprague Dawley rats, aged 8 weeks, were purchased from Shanghai SLAC Laboratory Animal, Co, Ltd. (Shanghai, China). 12 rats were randomly selected and placed in a prone position after being anesthetized by administering intraperitoneal injections of 90 mg/kg ketamine and 10 mg/kg xylazine. Under fluoroscopic guidance, the tail skins of 12 rats were disinfected with ethanol and punctured using an 18-gauge needle from the dorsal side at the same disk. The needle was punctured through the center of the disk until the opposite side, rotated 180°, and held for 10 s [30]. The adenovirus (pAdeasy-EF1- hsa_circ_0007290-CMV-EGFP-N) was injected into 6 rats’ surgery disk 10 μl every 14 days [31]. The remaining 12 rats underwent no surgical intervention and served as the negative controls. 6 of them were also injected the adenovirus with the same frequency and dose while others accepted NC adenovirus injection. Post-surgery, the wound was covered with gauze, and a standard postoperative procedure was performed. After 4 weeks, the rats were euthanized using an overdose of chloral hydrate.

### MRI and micro-CT evaluation

The collected caudal spines of Sprague-Dawley (SD) rats were preserved in a 4% paraformaldehyde solution for 48 h and subjected to MRI (MR5300, Philips, Netherlands) and micro-CT (Skyscan1072, Belgium). The water content of lumbar IVD was measured on sagittal T2W1 MRI. IVD height was measured using the ImageJ software and expressed as the disk height index (DHI) using a previously described method [32]. Images and 3D reconstructions of micro-CT images were generated using the corresponding software.

### Nucleic acid electrophoresis

Genomic DNA (gDNA) was purified from human NPCs seeded in 6-well plates (1 × 10^5^ cells per well) using an Ultrapure RNA kit (CWBIO, Beijing, China) according to the manufacturer’s instructions. cDNA was generated from the total RNA described below. gDNA and cDNA were amplified by PCR using the MasterMix (Yeason, Shanghai, China) and specific primers for FUNDC1, circFUNDC1, and β-actin. Electrophoresis was carried out in 2% agarose gel by loading 1 μg each of gDNA, cDNA, and PCR products, and DNA molecular marker (Novizan, Nanjing, China).

### Fluorescence in-situ hybridization (FISH)

The Cy3-labeled specific probes for detecting circFUNDC1 were designed and synthesized by RiboBio (Guangzhou, China) (Table S3). Cultured NPCs and tissues were fixed with 4% paraformaldehyde for 10 min and washed three times with PBS. Cells were permeabilized with 0.1% Triton X-100 in PBS for 5 min and then washed three times with PBS. FISH signals were detected using a FISH kit (RiboBio, Guangzhou, China), according to the manufacturer’s guidelines. Briefly, samples were incubated with circFUNDC1 probes overnight at 37 °C, followed by washing three times with washing buffer at 42 °C. Nuclei were stained with 4, 6-diamidino-2-phenylindole (DAPI) (Life Technologies, Carlsbad, CA, USA) for 10 min at room temperature. Fluorescence images were acquired using a fluorescence microscope (BX51TRF; Olympus, Tokyo, Japan).

### ASOs, siRNAs, shRNAs, vector construction, and adenovirus overexpression

ASOs, siRNAs, and shRNAs targeting circFUNDC1 and FUNDC1, and miRNA mimics were designed and synthesized by RiboBio (Guangzhou, China) (Table S3). Synthetic miRNA mimics the function the endogenous miRNA counterparts. Lipofectamine iMax (Invitrogen, Carlsbad, CA, USA) (1 µl per 105 cells) was used to transfect siRNAs and miRNA mimics to human NPCs seeded in 6 or 12-well plates. To generate the FUNDC1 overexpression plasmid (pEnCMV-FUNDC1), the FUNDC1 cDNA was cloned into the pEnCMV vector, MiaolingBio (Wuhan, China). Adenovirus harboring the circFUNDC1 (pAdeasy-EF1- hsa_circ_0007290-CMV-EGFP-N) was generated and purified by Hanheng Biotechnology (Shanghai, China).

### RNA extraction, RNase R treatment, and RT-qPCR

Total RNA was isolated from human nucleus pulposus or cultured NPCs using TRIzol reagent and the RNA extraction kit (Accurate Biotechnology, Hunan, China) according to the manufacturer’s instructions. 5 µg total RNA was incubated with or without 3 U/µg RNase R (Epicentre Technologies, Madison, WI, USA) for 15 min at 37 °C to degrade linear RNAs. cDNA was synthesized from 1 µg of total RNA using Evo M-MLV RT Kit Mix for qPCR (Accurate Biology, Hunan, China). RT-qPCR was performed with Hieff® qPCR SYBR Green Master Mix (Yeasen, Shanghai, China) and specific primers using an ABI 7500 Sequencing Detection System (Applied Biosystems, Foster City, CA, USA) according to the manufacturer’s protocols. The relative expression levels of the qPCR products were calculated using the 2-ΔΔCt method. Individual gene expression levels were normalized to those of β-actin for mRNA analysis.

### Dual-luciferase reporter assay

To assess whether circFUNDC1 binds to its predicted miRNA targets and becomes degraded in the process, an experimental system was established to measure circFUNDC1 activity on the FUNDC1 promoter. We generated plasmids containing dual-luciferase reporters based on the pGL3-Firefly luciferase-Renilla luciferase vector (Tsingke, Hangzhou, China), where firefly luciferase expression is driven by either the wildtype or mutated FUNDC1 promoter sequence (2000 bp upstream of the transcription start site). Renilla luciferase expression remained unchanged, serving as a normalizing control.

HEK-293T cells seeded in 24-well plates (2 × 10^4^ cells per well) were transfected with various combinations of the following plasmids: dual-luciferase plasmids driven by the wildtype/mutated FUNDC1 promoter, p-CMV-CDK9 overexpression or control pCMV plasmid (RiboBio, Guangzhou, China), and infection with Ad-circFUNDC1 or control adenovirus. After 48 h, luciferase activity with a microplate reader (BioTek, VT, USA) using the Dual-Luciferase Reporter Gene Assay Kit (Beyotime, Shanghai, China) according to the manufacturer’s protocols. Firefly luciferase activity in individual samples was normalized to Renilla luciferase activity. The relative luciferase activity was determined by comparing individual samples to the sample transfected with the dual-luciferase reporter only.

### Western blotting

NPCs were seeded in 6-well plates (1×10^5^ cells per well) and received various treatments before being harvested. Cells were lysed in RIPA lysis buffer (Fudebio, Hangzhou, China) with 1 mM phenylmethanesulfonylfluoride (Fudebio, Hangzhou, China), and total protein in the lysates was quantified with a bicinchoninic acid analysis kit (Beyotime, Shanghai, China). Total proteins (20 µg per well) were electrophorized on 10 or 12% SDS-polyacrylamide gels (Fudebio, Hangzhou, China) along with the GoldBand Plus 3-color Regular Range Protein Marker (Yeason, Shanghai, China). Resolved bands were electroblotted onto polyvinylidene fluoride membranes (Yeason, Shanghai, China) and blocked with 5% skimmed milk. Then the membranes were incubated overnight at 4 °C with specific antibodies including β-actin (1:2000, Cell Signalling Technology), FUNDC1, P21, P16, collagen II, Sox9 (all at 1:1000, Abcam), TOM20(1:1000, Proteintech), CDK9 (1:1000, Proteintech). After washed with TBS containing 0.1% Tween 20, membranes were incubated with secondary antibodies conjugated to horseradish peroxidase (1:5000, Fudebio, Hangzhou, China) at room temperature for 1 h. Protein bands were detected using FDbio-Femto ECL (Fudebio, Hangzhou, China) substrates and a chemiluminescence apparatus (Vilber, France).

### Flow cytometry

NPCs infected with Ad-circFUNDC1 were seeded in 6-well plates (1×105 cells per well) and treated with 200 μM H_2_O_2_ for 48 h. Cells producing reactive oxygen species (ROS) were stained using the Reactive Oxygen Species Assay Kit (Beyotime, Shanghai, China) according to the manufacturer’s protocols, and detected using a BD LSRFortessa cell analyzer (BD Biosciences, USA). The FlowJo software was used to evaluate the results.

### Hematoxylin-Eosin (HE), Alcian blue, and Safranin-O/fast green staining

Human nucleus pulposus specimens were fixed with 4% paraformaldehyde for 48 h. The samples were decalcified in 10% EDTA for 3 weeks and then processed, paraffin-embedded, and sectioned at a thickness of 5 μm. Sections were oven-dried at 60 °C overnight. Sections were then stained with 0.1% Safranin O and 0.001% fast green solution or Alcian blue solution, pH 2.5 (Thermofisher, MA, USA) to reveal the nucleus pulposus. An HE staining kit (Solarbio, Beijing, China) was used according to the manufacturer’s protocols. Images were acquired using the CX33TRF microscope (Olympus, Tokyo, Japan).

### High-density mass culture

1.5 × 10^5^ NPCs were resuspended in 10 μl of DMEM (Gibco-Thermo Fisher Scientific, Waltham, MA, USA) containing 10% FBS and seeded as a single high-density mass in the middle of a 24-well plate and incubated at 37 °C in 5% CO_2_. The seeded mass became adherent in 2 h. Then 0.5 ml DMEM containing 10% FBS was added to fill each well and refreshed every day. After 7 days, high-density masses were stained with Alcian blue using an Alcian Blue Stain Kit, pH 2.5 (SolarBio, Beijing, China) according to the manufacturer’s instructions.

### Molecular docking RNA

The crystal structure of CDK9 was obtained from the Protein Data Bank. The Amber14SB force field was used for energy optimization. Docking between CDK9 and the nucleic acid interaction region in circFUNDC1 was carried out by Rosetta, using the Monte Carlo algorithm for a comprehensive conformational search. During the low-resolution sampling stage, the backbone atoms and amino acids side-chain centroids were used to construct a model of the protein and to determine which conformations were retained. In the high-resolution optimization stage, all heavy and polar hydrogen atoms were restored, and the Monte Carlo algorithm was used to optimize and insert the side chains. At this stage, a more detailed and complex all-atom potential function was used for scoring. Finally, a composite scoring function and binding mode were used to select the most reasonable docking result.

### RNA Pull-down assay

1 × 10^7^ NPCs were collected, lysed, and sonicated. The Pierce^TM^ Magnetic RNA-Protein Pull-Down Kit (Thermo Fisher Scientific) was used for the pull-down assay. Beads were washed twice with 20 mM Tris before use. Biotinylated full-length circFUNDC1 or the kit-provided negative control (RiboBio, Guangzhou, China) was incubated with 50 μl of streptavidin magnetic beads in RNA capture buffer at room temperature for 30 min. Then the NPC lysates were incubated with biotinylated RNA bound to streptavidin beads at 4 °C overnight. Beads were washed four times with wash buffer and the bound proteins were eluted with Biotin Elution Buffer for 15 min at 37 °C. The eluted proteins were analyzed by Western blotting. The RNA was analyzed by RT-qPCR.

### Silver staining

Silver staining was performed using a Fast Silver Stain Kit (Beyotime, Shanghai, China) according to the manufacturer’s instructions. Briefly, the protein eluates obtained from the RNA pulldown assay was denatured at 95 °C in 5X sample loading buffer (Fudebio, Zhejiang, China) for 5 min and loaded onto an SDS-polyacrylamide gel for electrophoresis. The gels were fixed with a stationary liquid (50 mL ethanol, 10 mL acetic acid, and 40 ml double distilled H_2_O) for 1 h. Then the gels were washed with 30% ethanol for 10 min and washed twice with double distilled H_2_O. The gels were incubated with silver staining buffer for 10 min and washed twice with double-distilled H_2_O. Gels were stained with a chromogenic agent until clear bands were visible, after which they were washed with the corresponding elimination agent.

### Mass spectrometry analysis

Specific bands from the silver-stained gel were excised and cut into small pieces, then destained with 50% acetonitrile in 50 mM ammonium bicarbonate (NH4HCO3). The destained gel pieces were dehydrated in 100% acetonitrile for 5 min and incubated with 10 mM Tris (2-carboxyethyl) phosphine hydrochloride at 37 °C for 30 min. Then, the gel was again dehydrated with 100% acetonitrile and incubated with 25 mM iodoacetamide at room temperature for 30 min in the dark. Gel pieces were washed with 50 mM NH4HCO3 and dehydrated with 100% acetonitrile. Finally, the gel piece was rehydrated and digested with 2 μg trypsin in 50 mM NH_4_HCO_3_ at 37 °C overnight. After digestion, the peptides were extracted from the gel pieces using 50% acetonitrile/0.1% formic acid. The extracted peptides were dried in a speed vacuum concentrator and resuspended in 0.1% formic acid for liquid chromatography-tandem mass spectrometry (LC-MS/MS). Peptide samples were dissolved in mobile phase A (0.1% formic acid) and separated using an EASY nLC-1200 (Thermo Scientific, MA, USA). The nano-liquid chromatography gradient was maintained at a constant flow rate of 400 nL/min and comprised of an increase from 2% to 7% mobile phase B (0.1% formic acid in 80% acetonitrile) over 1 min, 7%–35% for 35 min, 35%–55% for 9 min, climbing to 100% in 7 min, and held at 100% for the last 8 min. The isolated peptides were subjected to a nano source, followed by Q Exactive HF-X mass spectrometry. The electrospray voltage applied was 2.0 kV, and intact peptides were detected in the Orbitrap at a resolution of 60,000. Peptides were then selected for MS/MS using an NCE setting of 27, and the fragments were detected using Orbitrap at a resolution of 30,000. Mass Spectra were acquired in data-dependent scan mode, including the selection of the 20 most abundant precursor ions in each MS spectrum for MS/MS analysis with 20 s dynamic exclusion. Mass spectra were processed and searched against the UniProt protein database using Proteome Discoverer (version 2.4, Thermo Scientific, MA, USA). Trypsin/P (or other enzymes if any) was specified as the cleavage enzyme, allowing for up to two missing cleavages.

The mass tolerance allowed for the precursor ions was 10 ppm, whereas the mass tolerance of fragment ions was set to 0.01 Da. Carbamidomethyl on Cysteine was specified as a fixed modification, whereas oxidation on methionine and acetyl on the protein N-terminal was specified as a variable modification. The peptide confidence was set at high.

### RNA immunoprecipitation (RIP)

1 × 10^7^ NPCs were transfected with miRNA mimics for miR-5002-3p, miR-502-5p, miR-6778-3p, or miR control. After 48 h, RIP was performed using a Magna RIP RNA-Binding Protein Immunoprecipitation Kit (Millipore, Billerica, MA, USA) according to the manufacturer’s protocol. Briefly, the cells were lysed in a complete RIP lysis buffer. The cell lysates were incubated with 5 μg of anti-Ago2 or control IgG antibody at 4 °C overnight. Ago2 is a protein used to detect complex formation between miRNAs and circRNAs. Total RNA was then isolated for the detection of circFUNDC1 by RT-qPCR as described above.

### CUT & Tag chromatin immunoprecipitation

The CUT & Tag assay was performed using a CUT & Tag assay kit (Novizan, Nanjing, China). NPCs were harvested, counted, and centrifuged for 3 min at 600×g at room temperature. 1 × 10^5^ cells were washed twice in 500 μl wash buffer by gentle pipetting. 10 µl of activated Concanavalin A-coated magnetic beads were added to each sample and incubated at RT for 10 min. Cells were then sequentially incubated the anti-CDK9 antibody (Proteintech), Goat anti-Rabbit IgG secondary antibody (1:5000, Fudebio, Hangzhou, China), and Hyperactive PG-TN5 / PA-TN5 Transposon and then fragmented. The fragmented DNA was extracted from the samples and amplified by PCR. The CUT & Tag libraries were constructed using HyperactiveTM In-Situ ChIP Library Prep Kit (TD901, Vazyme, China) and sequenced on an Illumina NovaSeq platform, and 150-bp paired-end reads were generated. Fastp v 0.20.0 was used to remove the adapter and low-quality reads. Paired-end reads were aligned to hg38 using Bowtie2 version 2.3.4.3 with options: --end-to-end –sensitive. Duplicated reads were removed using Picard v2.18.17 with this parameter: REMOVE_DUPLICATES = true. Peak calling used SEACR v1.3 with a threshold: of 0.01. Scatterplots, correlation plots, and heatmaps were displayed using deepTools v2.27.1. Annotation of peaks was performed using an R package ChIPseeker v1.12.1. MEME-ChIP v 5.0.5 was used to search for the binding site. Peaks with M-value > 0.2 and *P*-value < 0.05 are defined as specific peaks.

### Immunohistochemistry (IHC)

Human nucleus pulposus were fixed with 4% paraformaldehyde for 48 h, decalcified in 10% EDTA, embedded in paraffin, and sectioned at 5 μm. For IHC, the slides were incubated with sodium citrate antigen retrieval solution (Solarbio, Beijing, China) at 60 °C overnight. Endogenous peroxidase activity was blocked by incubation with 3% hydrogen peroxide for 10 min. Slides were washed three times with PBS and blocked with 5% bovine serum albumin (BSA) in PBS for 1 h at room temperature. Slides were incubated with antibodies against TOM20, FUNDC1, P16, P21, collagen II, and aggrecan at 4 °C overnight. After washing three times with PBS, slides were incubated with HRP-conjugated goat anti-rabbit secondary antibodies (ZSGB-Bio, Beijing, China) for 1 h at room temperature and were then washed 3 times with PBS. The slides were developed with diaminobenzidine tetrahydrochloride (ZSGB-Bio, China) for three min at room temperature. Images were acquired using a 40x objective microscope (CX33TRF, Olympus, Tokyo, Japan).

### Senescence-associated β-galactosidase (SA-β-Gal) staining

NPCs infected with Ad-circFUNDC1 were seeded in 6-well plates (1 × 10^5^ cells per well) and treated with 200 μM H_2_O_2_ for 7 days. SA-β-Gal staining was performed with the SA-β-Gal staining kit (Beyotime, Shanghai, China). Briefly, after washing twice with PBS, NPCs were fixed with 4% paraformaldehyde and 0.2% glutaraldehyde for 15 min at room temperature. Fixed cells were washed three times with PBS and incubated with 1 mL SA-β-Gal staining solution at 37 °C overnight. The next day, the cells were washed twice with PBS and 70% ethanol.

### Confocal microscopy

NPCs were treated with different conditions and the production of ROS was detected using a Reactive Oxygen Species Assay Kit (Beyotime, Shanghai, China) according to the manufacturer’s protocols. Images were captured using an inverted laser confocal microscope (Olympus Corporation FV3000).

### Statistical analysis

Statistical analyses were performed using the GraphPad Prism software (version 9.0.0). Statistical significance was determined using unpaired Student’s *t*-test, paired Student’s *t*-test, one-way ANOVA, two-way ANOVA, Kruskal–Wallis test, and Mann–Whitney U test. Results were considered statistically significant at *P* < 0.05.

## Results

### circFUNDC1 is markedly downregulated in end-stage IVDD

**Fig. 1:**
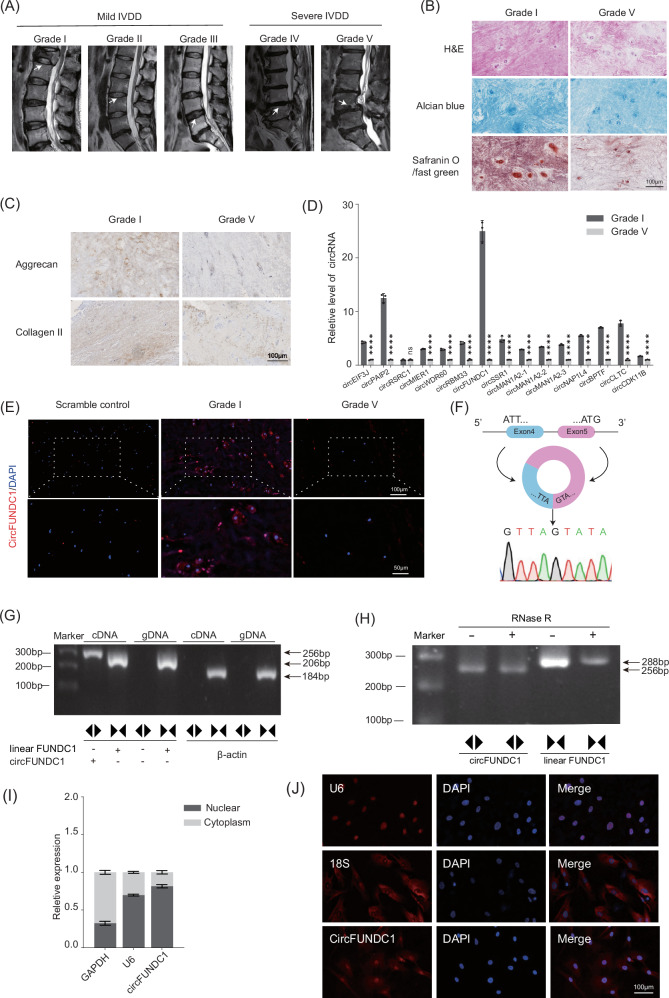
circFUNDC1 is markedly downregulated in end-stage IVDD and predominately located in the nuclei of NPCs. **A** MRI evaluation of patients with grade I to grade V IVDD. **B** Histological assessment of grade I and grade V nucleus pulposus (scale bar = 100 μm). **C** Representative images of aggrecan and collagen II expression in the nucleus pulposus detected by IHC (*n* = 3 donors, scale bar = 100 μm). **D** qPCR analysis of the relative levels of the top 15 RNA Pol II-associated nuclear circRNAs in grade V nucleus pulposus compared to grade I (data represented mean ± SEM from 3 independent experiments, ns *p* > 0.05, ****p < 0.0001 vs control or as indicated by the student’s t-test). **E** Representative images of circFUNDC1 expression in grade I and V nucleus pulposus detected by a circFUNDC1-specifc or scrambled FISH probe. The nuclei were stained with DAPI. (*n* = 3 donors scale bars = 50–100 μm). **F** Schematic showing the circularization of FUNDC1 mRNA via back-splicing of exon 4 and part of exon 5 to form circFUNDC1. Sanger sequencing confirmed the detection of the back-splice junction. **G** PCR analysis of gDNA and cDNA from NPCs using divergent and convergent primer pairs (indicated by the directions of arrows) confirmed the absence of genomic rearrangement in the FUNDC1 gene. Expected PCR product sizes were 256 bp with divergent primers detecting linear FUNDC1 mRNA and 206 bp with convergent primers detecting circFUNDC1. Divergent and convergent primers for β-actin were used as a control. **H** RNase R treatment prior to PCR analysis demonstrated circFUNDC1, but not linear FUNDC1 mRNA, is resistant RNase R digestion. **I** Nuclear/cytoplasmic distribution of circFUNDC1 detected by RT-qPCR analysis of total RNAs isolated from the nuclear or cytoplasmic fraction of NPCs (*n* = 3 donors). GAPDH (cytoplasmic dominant) and U6 (nuclear dominant) were used as controls. **J** Representative images of circFUNDC1 expression in nucleus and cytoplasm detected by a circFUNDC1-specifc FISH probe. The nuclei were stained with DAPI. 18S (cytoplasmic dominant) and U6 (nuclear dominant) were used as controls. (*n* = 3 donors, scale bars = 100 μm).

IVDD is classified into five clinical grades (Fig. 1A). Grades I to III indicate mild degeneration, based on the degree of disc herniation. Grades VI and V represent severe degeneration, often causing significant nerve compression (Fig. S1A) [33, 34]. When comparing Grade I and Grade V clinical samples used in this study, Grade I NPCs were regularly spaced, while Grade V NPCs were often fused together, with a marked reduction in proteoglycans and ECM components (Fig. 1B&C). Using qPCR, we examined the expression levels of the top 15 nuclear circRNAs associated with RNA polymerase II [27] and found that circFUNDC1 was the most downregulated in Grade V NPCs compared to Grade I (Fig. 1D). The downregulation of circFUNDC1 in Grade V NPCs was confirmed by FISH (Fig. 1E) and by quantifying positive staining cells in these samples (Fig. S1B).

### circFUNDC1 is predominately located in the nuclei of NPCs

The circRNA database (circBase) predicts that circFUNDC1 is formed by back-splicing of the FUNDC1 mRNA between exon 4 and exon 5. The mature circFUNDC1 is 403 nucleotides in size. The identity of circFUNDC1 isolated from NPCs was confirmed by Sanger sequencing using specific primers that detected the junction derived from the back-splicing of the 3’ end of exon 4 to the 5’ splice site of exon 5 (Fig. 1F). To rule out the possibility of genomic rearrangements that could yield similar sequencing results, we separately prepared and analyzed genomic DNA and cDNA from NPCs using PCR with divergent and convergent primers that will either detect the linear or circular form of FUNDC1 mRNAs, yielding PCR products of 288 or 256 bp, respectively. Results showed the presence of both the linear FUNDC1 mRNA and the circularized circFUNDC1 in the cDNA sample as expected, but not in the genomic DNA, indicating no genomic rearrangement (Fig. 1G). Furthermore, because circular RNAs, not linear RNAs, are resistant to RNase R digestion, the PCR analysis was repeated using total RNAs pretreated with RNase R. Results showed that RNase R treatment reduced only the PCR product derived from the linear FUNDC1 mRNA (288 bp), not the circular form (256 bp) (Fig. 1H). We next investigated the subcellular location of circFUNDC1. qPCR analysis of total RNAs isolated from the nuclear or cytoplasmic fraction revealed that circFUNDC1 was predominately found in the nucleus (Fig. 1I). A similar result was also observed using FISH analysis (Fig. 1J). These results confirmed the presence of predominately nuclear-located circFUNDC1 in NPCs.

### circFUNDC1 is involved in the homeostasis of ECM

**Fig. 2:**
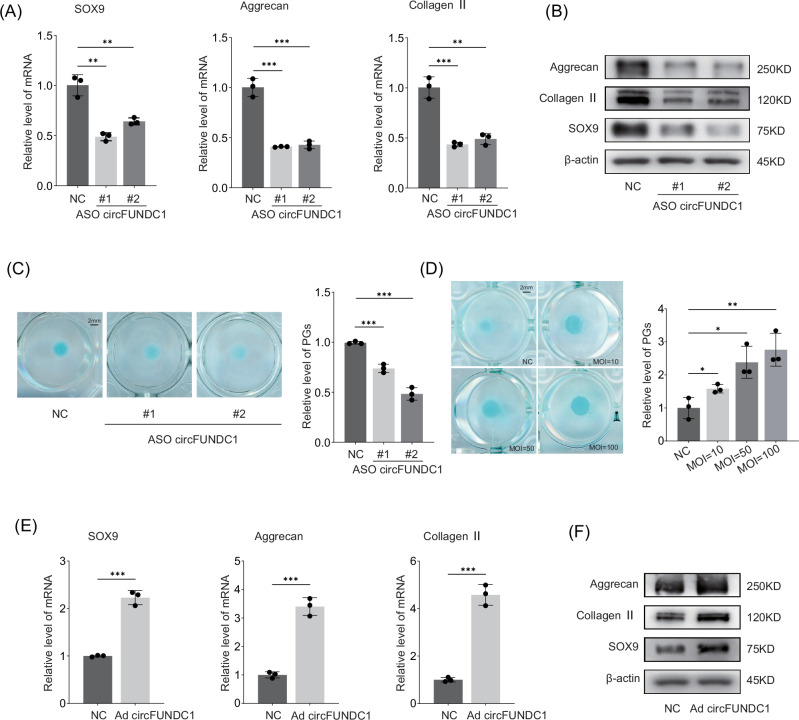
circFUNDC1 is involved in the homeostasis of ECM. **A** qPCR and (**B**) Western blot analysis of Sox9, aggrecan, and collagen II expression in NPCs transfected with scrambled (NC) or anti-sense oligos (ASO) #1 & #2 for circFUNDC1. **C** Representative images of Alcian blue staining of high-density cultures of NPCs transfected with ASO circFUNDC1 (scale bar =2 mm). Staining intensity of proteoglycans (PGs) was quantified and normalized to the control. **D** Representative images of Alcian blue staining of high-density cultures of NPCs infected with adenovirus expressing circFUNDC1 (Ad circFUNDC1) or control virus (NC) at different multiplicity of infection (MOI). Staining intensity of proteoglycans (PGs) was quantified and normalized to the control (scale bar =2 mm). **E** qPCR and (**F**) Western blot analysis of Sox9, aggrecan, and collagen II expression in NPCs infected with Ad circFUNDC1 or control virus. Data were representative images of similar results obtained from 3 different donors (**B**, **C**, **D**, and **F**) or presented as the mean ± SEM from 3 independent experiments (**A** and **E**). (*p < 0.05, **p < 0.01, ***p < 0.005 vs control or as indicated by Student’s *t*-test).

We next examined the role of circFUNDC1 in IVDD. Using two antisense RNA oligos (ASOs), we knocked down circFUNDC1 expression in NPCs (Fig. S1C). This depletion led to a reduction in both the mRNA and protein levels of anabolic factors Sox9, aggrecan, and collagen II after 48 h (Fig. 2A&B). Additionally, knockdown of circFUNDC1 suppressed proteoglycan production in high-density NPC cultures (Fig. 2C). We then overexpressed circFUNDC1 in NPCs via adenovirus infection at a multiplicity of infection (MOI) that did not affect cell growth (Fig. S1D). The levels of ectopically expressed circFUNDC1 were assessed using qPCR (Fig. S1E), and specific probes were employed to verify the presence of circularized RNA instead of the linear form (Fig. S1F). To demonstrate that adenovirus can function effectively in cells for an extended period, qPCR was performed (Fig. S1G). In high-density NPC cultures, overexpression of circFUNDC1 resulted in a dose-dependent increase in proteoglycan production (Fig. 2D). Moreover, overexpressing circFUNDC1 elevated the mRNA and protein levels of anabolic factors Sox9, aggrecan, and collagen II (Fig. 2E&F). These results indicate that circFUNDC1 is required to maintain the levels of ECM components and SOX9 produced by NPCs.

### circFUNDC1 interacts with the transcriptional regulator CDK9

**Fig. 3:**
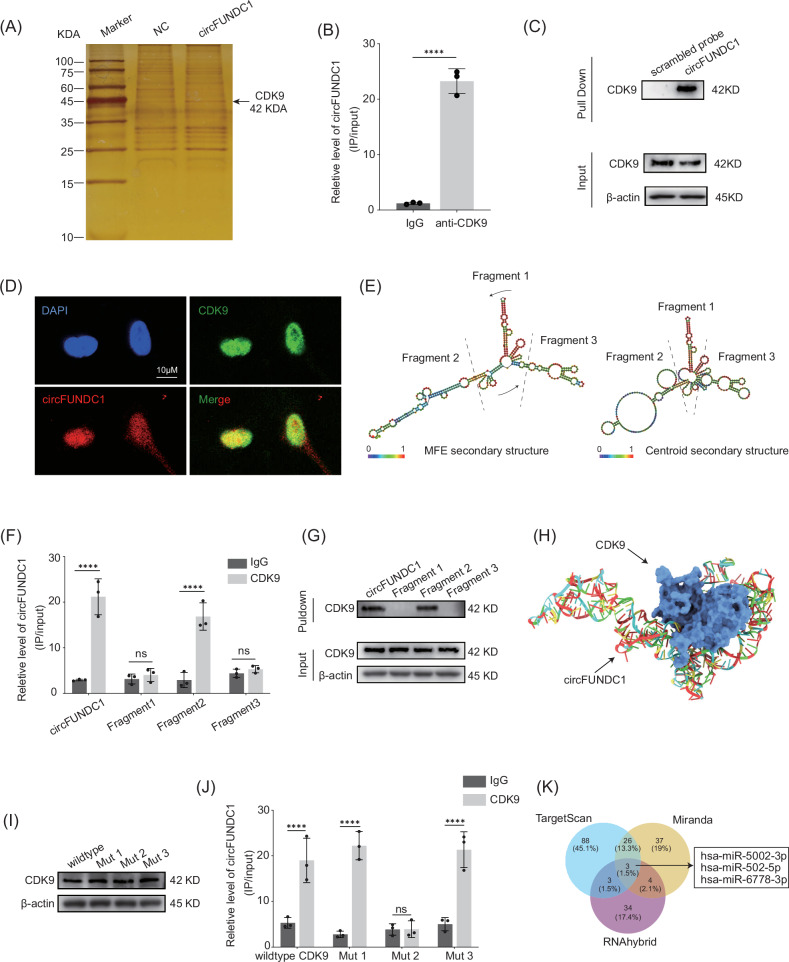
circFUNDC1 interacts with the transcriptional regulator CDK9. **A** Silver staining of eluates from an RNA pull down assay using biotinylated circFUNDC1 detected a prominent band at 42 KDA, later identified by mass spectrometry as CDK9. **B** qPCR detection of circFUNDC1 co-purified by RNA immunoprecipitation (RIP) using a rabbit anti-CDK9 antibody. Rabbit IgG was used as a control. **C** Western blot detection of CDK9 pulled down by biotinylated circFUNDC1 or scrambled probe. **D** Colocalization of circFUNDC1 and CDK9 detected by FISH and anti-CDK9 antibody, respectively. Nuclei were stained with DAPI (scale bar = 10 μm). **E** Diagrams illustrated the arbitrary division of circFUNDC1 into 3 fragments based on the most stable secondary structures (MFE and Centroid methods) predicted by RNAfold. Arrows indicated the 5’-3’ direction. **F** qPCR detection of wildtype or circFUNDC1 fragments from RIP using an anti-CDK9 antibody or IgG. **G** RNA pulldown assay using wildtype circFUNDC1 or fragmented circFUNDC1 and Western blot detection of CDK9. **H** CDK9 and circFUNDC1 interaction modeled by molecular docking. **I** Western blot of CDK9 mutants in transfected NPCs. **J** qPCR detection of circFUNDC1 from RIP of NPCs expressing wildtype or mutant CDK9. **K** Venn diagram showing the 3 overlapping candidates predicted by miRanda, RNAhybrid, and TargetScan to be the miRNA targets of circFUNDC1. Data were presented as the mean ± SEM from three independent experiments (**B**, **F**, and **I**). (ns *p* > 0.05, ****p < 0.001 vs control or as indicated by Student’s *t*-test).

Next, we investigated the mechanism by which circFUNDC1 regulates ECM production. Unlike most cytoplasmic circRNAs that act as sponges for miRNAs to negate their effects on mRNA translation [35, 36], circFUNDC1 is predominately localized in the nucleus. We postulated that circFUNDC1 may function as a transcriptional regulator by interacting with other nuclear factors. Mass spectrometry analysis of RNA pull-down samples using circFUNDC1 as bait identified CDK9 as a major circFUNDC1 binding partner, as seen in the silver stain (Fig. 3A). CDK9 is a transcriptional regulator important for the unpausing of RNA Polymerase II during transition of the initiation to elongation stage of transcription [37, 38]. The interaction between circFUNDC1 and CDK9 was confirmed by immunoprecipitation using an anti-CDK9 antibody followed by qPCR detection of circFUNDC1 (Fig. 3B), and by RNA pull-down assay using a specific circFUNDC1 probe followed by CDK9 Western blot (Fig. 3C). Additionally, immunofluorescence and FISH confirmed the co-localization of circFUNDC1 and CDK9 in the nucleus as expected (Fig. 3D).

We next investigated the molecular basis of circFUNDC1-CDK9 interaction. Using a predictive algorithm (RNAfold), we divided the secondary structure of circFUNDC1 into 3 fragments (Fig. 3E, Table S1) and generated individual expression plasmids. Fragments 2 and 3 were derived from contiguous circFUNDC1 sequences that form stem-loop structures. Fragment 1 was derived from joining two opposing sections within circFUNDC1, while it was predicted to retain a similar secondary structure as in the native circFUNDC1 (Fig. S1H). When these RNA fragments were expressed as circular forms in NPCs, only fragment 2, corresponding to nucleotides 55-200 in circFUNDC1, was able to interact with CDK9, as revealed by immunoprecipitation (Fig. 3F) and RNA-immunoprecipitation (Fig. 3G). These results indicate that the stem-loop structure formed by fragment 2 in circFUNDC1 is crucial for CDK9 interaction.

To further investigate the structural basis of circFUNDC1 and CDK9 binding, the Rosetta RNA-protein docking algorithm was used to model the interaction between CDK9 and the full length of circFUNDC1. Results identified several nucleotides in circFUNDC1 that could potentially form hydrogen bonds with the amino acid residues in CDK9 (Fig. 3H, Table S2). The contacting nucleotides and residues between circFUNDC1 and CDK9 can be grouped into four major clusters (Fig. S2A-D). Since fragment 2 of circFUNDC1 is important for CDK9 binding, we then performed alanine-scanning mutagenesis on selected amino acid residues in clusters A and C to generate CDK9 Mut 1, 2, and 3 (Fig. S2E). While these mutations did not affect the protein expression of CDK9 mutants (Fig. 3I), Mut 2 lost its ability to bind circFUNDC1 (Fig. 3J), indicating the contacting residues in Mut 2 (aa 250-260) are important for binding circFUNDC1. Taken together, these results strongly suggest that interaction occurs between nucleotides in fragments 2 of circFUNDC1 and aa 250-260 in CDK9.

Given that only a minor fraction of circFUNDC1 is found in the cytoplasm, we predicted it would likely not function as a miRNA sponge. To confirm this, we identified the top three miRNA candidates (miR-5002-3p, miR-502-5p, and miR-6778-3p) as potential targets of circFUNDC1 using three different miRNA target prediction algorithms (miRanda, RNAhybrid, and TargetScan) (Fig. 3K). When overexpressed, these miRNAs did not affect luciferase expression driven by the FUNDC1 promoter (Fig. S3A) or the levels of endogenous circFUNDC1 as determined by RIP (Fig. S3B), suggesting that circFUNDC1 does not act as molecular sponge for these three top miRNA targets.

### circFUNDC1-CDK9 complex activates the FUNDC1 promoter

**Fig. 4:**
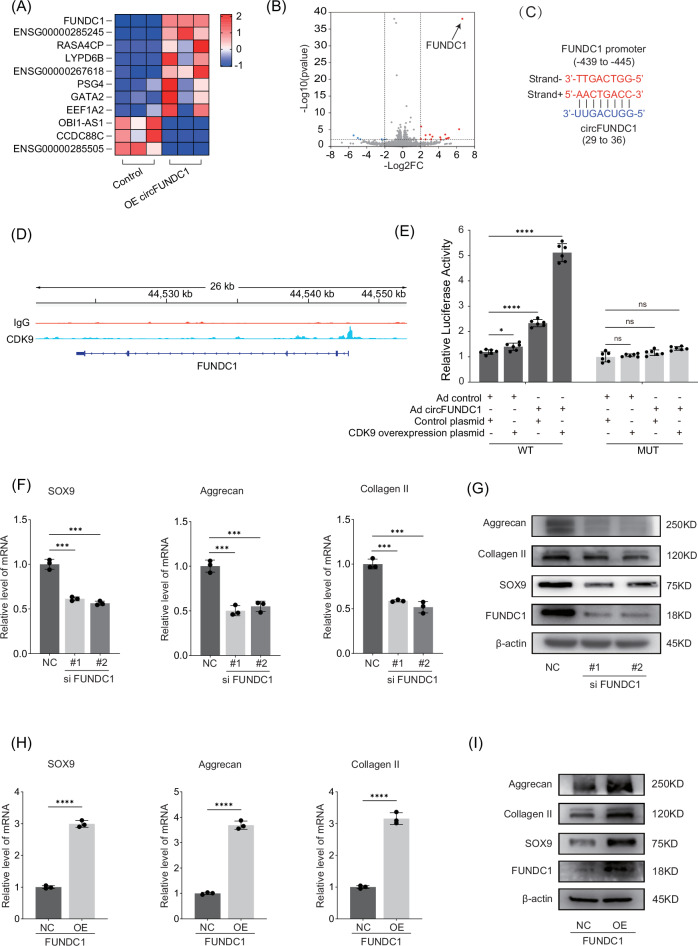
The overexpression of FUNDC1 is activated by the circFUNDC1-CDK9 complex and has a protective effect on NPCs. Heatmap map (**A**) and volcano map (**B**) of the eleven most up or down-regulated mRNAs in NPCs overexpressing circFUNDC1. **C** Complementary nucleotides between the FUNDC1 promoter (-439 to -445) and circFUNDC1 (29 to 36). **D** CUT&Tag chromatin immunoprecipitation detect high levels of CDK9 the FUNDC1 promoter. **E** Dual-luciferase reporter assay. NPCs were co-transfected with luciferase reporters driven by the wild-type or mutated FUNDC1 promoter, with or without empty vectors and vectors for circFUNDC1 and CDK9 overexpression. Levels of mRNAs (**F**) and proteins (**G**) of Sox9, aggrecan, and collagen II in NPCs transfected with scrambled or siRNA#1 and #2 against FUNDC1. **H** and proteins (**I**) of Sox9, aggrecan, and collagen II in NPCs transfected with empty plasmids or FUNDC1 expression plasmids. Data were representative images of similar results obtained from three different donors (**G** and **I**) or presented as the mean ± SEM from three independent experiments (**E**, **F**, and **H**). (ns *p* > 0.05, ****p < 0.001 vs control or as indicated by Student’s *t*-test).

We then explored the effects of the circFUNDC1-CDK9 interaction on transcription. RNA-Seq analysis revealed that overexpression of circFUNDC1 significantly upregulated 8 genes and downregulated 3 genes in NPCs (Fig. 4A&B). Notably, FUNDC1 mRNA was among the most upregulated, suggesting that ectopically expressed circFUNDC1 enhances FUNDC1 mRNA transcription. We hypothesized that this could be mediated by complementary sequences in circFUNDC1 and the FUNDC1 gene. Sequence alignment confirmed the presence of an 8-nucleotide complementary stretch in both the FUNDC1 promoter and circFUNDC1 (Fig. 4C). Given that circFUNDC1 and CDK9 interact, we examined whether binding to cirFUNDC1 recruits CDK9 to the FUNDC1 promoter. CUT & Tag chromatin immunoprecipitation confirmed high levels of CDK9 were found at the FUNDC1 promoter region (Fig. 4D). These findings suggest that circFUNDC1 and CDK9 form a ternary complex with complementary nucleotides in the FUNDC1 promoter. To test this, we constructed luciferase reporters driven by either wild-type or mutated FUNDC1 promoter sequences and assessed the effects of ectopic expression of circFUNDC1 and/or CDK9 on luciferase activity. The data showed that while circFUNDC1 alone slightly increased the luciferase activity, co-expression of circFUNDC1 and CDK9 markedly increased luciferase activity driven by the wild-type, but not the mutated, FUNDC1 promoter (Fig. 4E). These results indicate that circFUNDC1 and CDK9 act synergistically to bind and transactive the FUNDC1 promoter.

### The FUNDC1 protein and circFUNDC1 RNA promote mitophagy to protect the NPCs under oxidative stress

Given that FUNDC1 enhances cell survival during oxidative stress by facilitating mitochondrial autophagy, or mitophagy [29], we postulated that this could potentially offer a mechanism of protecting NPCs from interverbal disk degeneration. To explore this, we either depleted FUNDC1 using two specific siRNAs (Fig. S3C) or overexpressed FUNDC1 and examined the impacts on ECM production by NPCs. The results showed that FUNDC1 depletion reduced the mRNA and protein levels of anabolic factors Sox9, aggrecan, and collagen II (Fig. 4 F&G). In contrast, overexpression of FUNDC1 produced the opposite effects, enhancing the expression of these anabolic factors (Fig. 4H&I). These results confirmed that the anabolic effects of circFUNDC1 observed earlier (Fig. 2) were due to circFUNDC1’s ability to enhance FUNDC1 protein expression.

**Fig. 5:**
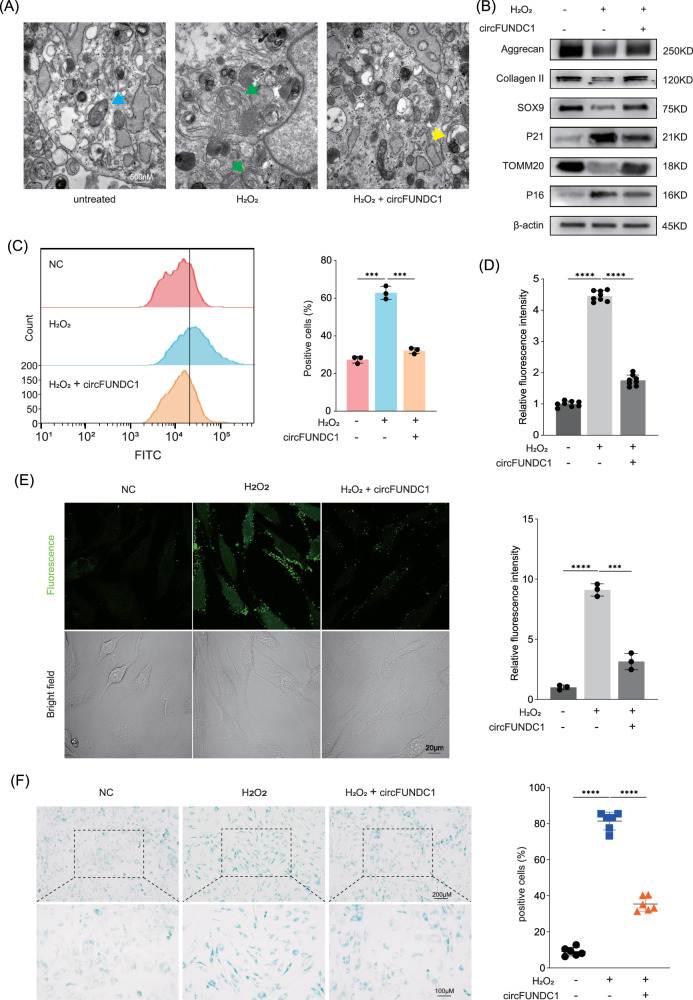
The FUNDC1 protein and circFUNDC1 RNA promote mitophagy to protect the NPCs under oxidative stress. **A** Representative transmission electron microscopy images of mitochondrial morphology in untreated (left), H_2_O_2_-treated (middle), and H_2_O_2_-treated + circFUNDC1 infected (right) NPCs (scale bar = 500 nm). Blue arrow: healthy mitochondria; green arrows: damaged mitochondria; yellow arrow: autophagosome. **B** Effects of circFUNDC1 overexpression on various proteins in untreated or H_2_O_2_-treated NPCs. **C** Fluorescence intensity of ROS in NPCs detected by flow cytometry. The cells with a fluorescence intensity over 1 × 10^4^ were counted as positive cells. **D** Relative fluorescence intensity of ROS in NPCs detected by a microplate reader. **E** Confocal images of NPCs stained with a fluorescent probe for ROS and the relative fluorescence intensity quantified (scale bar = 20 μm). **F** Representative images of β-Gal staining and of untreated or H_2_O_2_-treated NPCs overexpressing circFUNDC1 and quantification of percent positive cells (scale bar = 100-200 μm). Data were representative images of similar results obtained from three different donors(**A**, **B**, **E**, and **F**) or presented as the mean ± SEM from three independent experiments (**C** and **D**). (***p < 0.005, ****p < 0.001 vs control or as indicated by Student’s t-test).

We next investigated the role of circFUNDC1 in mitophagy. NPCs were treated with hydrogen peroxide to induce oxidative stress. As expected, untreated NPCs had healthy intact mitochondria with clearly visible membrane ridges. In contrast, peroxide-treated NPCs exhibited damaged and swollen mitochondria with disorganized cristae, indicative of impaired mitochondrial functions. Notably, overexpression of circFUNDC1 rescued these mitochondrial defects, and autophagosomes, the hallmark of mitophagy, were observed in the sample (Fig. 5A). Consistent with these findings, hydrogen peroxide treatment decreased the expression of anabolic factors and the mitochondrial marker TOMM20, while increasing the expression of senescence markers P16 and P21 (Fig. 5B). However, these effects were reversed by circFUNDC1 overexpression.

We then measured the effects of circFUNDC1 on reactive oxygen species (ROS) production in NPCs. Hydrogen peroxide-treated samples showed a significantly higher percentage of ROS-positive cells and elevated ROS levels (Fig. 5C-E). However, these effects were mitigated by circFUNDC1 overexpression. Since oxidative stress also promotes cellular senescence, we predicted that circFUNDC1 could protect NPCs from stress-induced senescence (Fig. 5F). Indeed, we observed that circFUNDC1 overexpression markedly reduced senescence of NPCs treated with hydrogen peroxide. To further investigate whether circFUNDC1 can reverse the deficiency of FUNDC1 and produce a therapeutic effect, western blotting, and qPCR experiments were performed (Fig. S3D, E). The results showed inhibiting FUNDC1 with siRNA would not block the effects of circFUNDC1. Together these results demonstrate a protective role of circFUNDC1 in reducing the oxidative damage in mitochondria of NPCs and preventing their senescence.

### circFUNDC1 prevents degeneration of human nucleus pulposus in ex-vivo culture

**Fig. 6:**
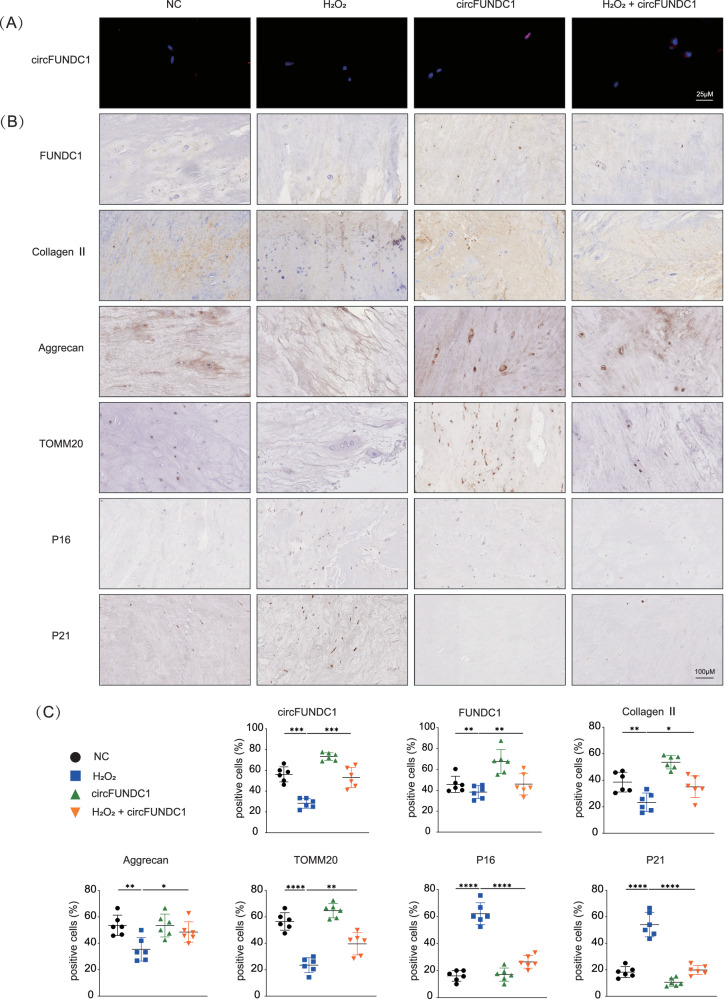
circFUNDC1 prevents degeneration of human nucleus pulposus in ex-vivo culture. **A** DAPI staining and a red FISH detection of circFUNDC1 in human nucleus pulposus infected with Ad circFUNDC1 (scale bar = 25 μm). **B** IHC detection of FUNDC1, collagen II, aggrecan, TOMM20, P16, and P21 expression in human nucleus pulposus (scale bar = 100 μm). Samples were collected from Grade I patients. **C** Percent positive cells for each marker were determined from 3 different fields. Data are representative images of similar results obtained from six different donors (**A** and **B**) or presented as the mean ± SEM (**C**). (*p < 0.05, **p < 0.01, ***p < 0.005, ****p < 0.001 vs control or as indicated by Student’s *t*-test).

First, we explored the expression level of FUNDC1 in human nucleus pulposus of Grade I to Grade V, which was consistent with our expectations (Fig. S3F). We next examined the protective effects of circFUNDC1 in an ex-vivo culture of human nucleus pulposus isolated from patients with grade I disease. Adenovirus-mediated overexpression of circFUNDC1 in the ex-vivo nucleus pulposus culture was confirmed by FISH (Fig. 6A). Overexpression of circFUNDC1 increased FUNDC1 proteins in the ex-vivo culture as expected (Fig. 6B). Moreover, circFUNDC1 significantly mitigated the degenerative effects of hydrogen peroxide treatment on the nucleus pulposus, as circFUNDC1 restored the expression of anabolic factors collagen II and aggrecan (Fig. 6C), and proteoglycan levels (Fig. S4A–C). Additionally, circFUNDC1 restored the mitochondrial maker TOMM20 expression- indicative of normal mitochondrial functions, and suppressed the expression of senescence markers P16 and P21- indicative of enhanced cell survival. Importantly, these immunohistological data were also confirmed by parallel Western blot and qPCR analyses (Fig. S4D–I). Taken together, these results rigorously confirmed the in vitro data above and strongly indicate that circFUNDC1 protects nucleus pulposus from oxidative stress by restoring mitochondrial functions, enhancing ECM production, and preventing senescence.

Next, we aimed to replicate the experimental effects in an animal model. Since circFUNDC1 is not conserved across species, we compared the sequences of circFUNDC1 and the FUNDC1 promoter region in rats (Sprague Dawley rats). We identified a similar nine-base sequence: CTGAAATCA in circFUNDC1 and CTGAAATGA in rats. This led us to hypothesize that circFUNDC1 might interact with the FUNDC1 promoter in rats through a similar mechanism. To investigate this, we overexpressed circFUNDC1 using adenovirus in rat nucleus pulposus cells and measured FUNDC1 expression levels (Fig. S5A). The results revealed no significant increase in FUNDC1 expression. Following this, we created an IVDD model using 24 eight-week-old rats. We injected adenovirus into the intervertebral disks of these model rats. CT scans indicated a significant reduction in intervertebral disc height four weeks post-modeling (Fig. S5B), while MRI images demonstrated a notable decrease in water content in the affected disks (Fig. S5C). Adenovirus-mediated overexpression of circFUNDC1 was confirmed through FISH, but no differences in proteoglycan levels were observed (Fig. S5D–F). We speculate that the mechanism of exogenous circFUNDC1 in rats may differ from that in humans, warranting further investigation into the underlying mechanisms.

## Discussion

In this study, we reported a novel protective role of the nuclear-located circFUNDC1 in the human nucleus pulposus. We showed for the first time an inverse correlation of circFUNDC1 levels with disease severity in clinical samples of IVDD, highlighting the importance of circFUNDC1 in maintaining intervertebral disc homeostasis. Our data conclusively demonstrated that circFUNDC1 delays intervertebral disc degeneration by mitigating the negative effects of oxidative stress on ECM production, mitochondrial deterioration, and cellular senescence, thus circFUNDC1 is a promising therapeutic target for IVDD. Our data revealed that circFUNDC1 is primarily distributed in the nucleus, with a small amount present in the cytoplasm. Since circRNAs do not have a 5’7-methylguanosine cap or a 3’ poly(A) tail, their degradation is usually caused by endonucleases [39]. The absence of endonucleases in the nucleus provides an environment where circFUNDC1 can persist longer and be better protected from degradation compared to circRNAs in the cytoplasm [40, 41].

We reported a novel interaction between circFUNDC1 and CDK9, a master regulator of transcriptional elongation in the nucleus. CDK9 interacts with its regulatory partner cyclin T, forming the positive transcription elongation factor-B (P-TEFb) that regulates elongation by phosphorylating RNA polymerase II [42–44]. The binding of circFUNDC1 to CDK9 was confirmed in reciprocating pull-down assays using either partner as baits. Molecular docking predicted the formation of numerous hydrogen bonds between circFUNDC1 and CDK9, and our data demonstrated that the stem-loop region (Fragment 2) in circFUNDC1 and residues 250-260 in CDK9 are particularly important for their interaction (Fig. 3).

Previous studies demonstrated the activity of CDK9 can be modulated by several mechanisms, either by small-molecule inhibitors [30, 45, 46], by interacting with other proteins [47], or by sumoylation [48]. However, this is the first time a circRNA has been shown to directly bind CDK9 and influence its activity on a specific gene. Our findings suggest that circRNAs may have a larger role in regulating transcription than previously thought and this has important clinical implications. Therapeutically targeting transcription of disease-causing genes has been a staple of modern molecular medicine. However, unlike conventional approaches using small molecules and protein biologics, circRNAs’ relative stability and simple but diverse structures could aid in developing therapeutics with better target specificity, affinity, biostability, and bioavailability.

We showed that circFUNDC1 recruits CDK9 to its parental gene FUNDC1 to increase FUNDC1 protein expression. Extensive research has been conducted on FUNDC1 since it was first reported as a mammalian receptor of mitophagy [29, 49], which is a type of autophagy that selectively removes damaged mitochondria. FUNDC1 is a mitochondrial out-membrane protein that interacts with the core autophagic machinery light chain 3 (LC3) for autophagosome formation [28]. The impaired ability of cells to remove damaged mitochondria resulted in excessive accumulation of ROS, which can damage cellular components and decrease ATP production, leading to cellular senescence [50, 51].

Due to their limited regenerative capacity, NPCs in the nucleus pulposus are susceptible to injury under long-term deforming stress, causing mitochondrial damage and ROS accumulation. Treatments focused on reducing ROS have been used in IVDD [52]. We investigated the roles of circFUNDC1 in NPCs subjected to oxidative stress and found that circFUNDC1 increases FUNDC1 expression, thereby inducing mitophagy and reducing senescence of NPCs, offering protection of the nucleus pulposus from ROS-mediated degeneration (Fig. 6). Thus, circFUNDC1 could be a promising RNA biologic to prevent, delay, or treat IVDD.

Limitations of the study include the lack of suitable animal models to fully explore the functions of circFUNDC1, as it is not conserved in other species. While experiments were conducted in rats, the specific mechanism of circFUNDC1 remains to be elucidated. Although we identified a novel ternary complex between circFUNDC1, CDK9, and the FUNDC1 promoter, the specific mechanism by which CDK9 promotes transcription of the FUNDC1 gene remains unclear. Future study is required to dissect the molecular mechanism of how circFUNDC1 halts senescence progression in NPCs under oxidative stress and prevents intervertebral disk degeneration.

In summary, this study demonstrates that circFUNDC1 delays IVDD by promoting mitophagy in NPCs challenged with ROS. Our findings provide new insight into the roles of circRNAs in human nucleus pulposus and present a novel therapeutic target for IVDD.

## Supplementary information

supplementary materials

Supplemental Material-WB

## Data Availability

The data supporting the findings of this study are available from the corresponding author upon reasonable request.
