# Supplementary material for: CircFUNDC1 interacts with CDK9 to promote mitophagy in nucleus pulposus cells under oxidative stress and ameliorates intervertebral disc degeneration
Source: Cell Death Dis. 2025 Feb 13;16(1):94. doi: 10.1038/s41419-025-07425-2 (PMC11825710; doi:10.1038/s41419-025-07425-2)
Supplement: Supplementary file 1 — supplementary materials [file 41419_2025_7425_MOESM1_ESM.docx]

Supporting information

**Figure S1**


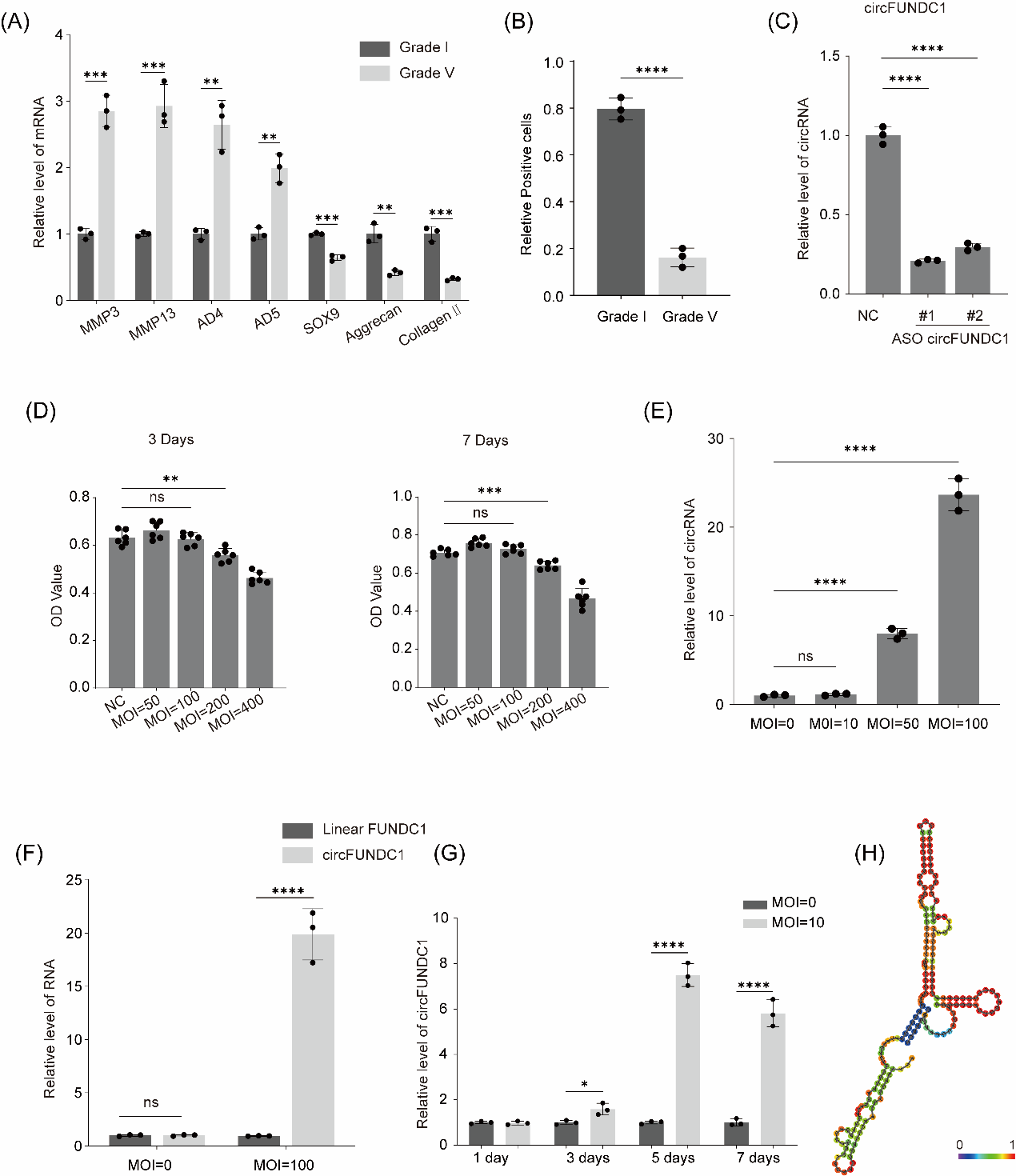


**Figure S1** **(A)** RT-qPCR showed decreased anabolic factors and increase MMP3 and 13 expressions in nucleus pulposus with end-stage IVDD. **(B)** FISH quantification of circFUNDC1-positive NPCs in samples with mild or end-stage disease. **(C)** The knockdown efficiency of two ASO against circFUNDC1 was determined by RT-qPCR. **(D)** Proliferation of NPCs infected with Ad circFUNDC1 at various MOI. **(E)** The overexpression efficiency of different MOIs with Ad circFUNDC1 was determined by RT-qPCR. **(F)** RT-qPCR using specific probes to detect the back-splicing junction showed the overexpression of circularized circFUNDC1 instead of its linear form in NPCs after adenovirus infection. **(G)** RT-qPCR showed adenovirus can function effectively in cells for 7days. **(H)** RNAFold prediction of circFUNDC1 fragment 1 to retain a similar secondary structure as it was within the wildtype circFUNDC1. Data are presented as the mean ± SEM from three independent experiments (A, B, C, D, E, F and G). (ns p>0.05, **p<0.01, ***p<0.005, ****p < 0.001 vs control or as indicated by the student’s t-test)

**Figure S2**


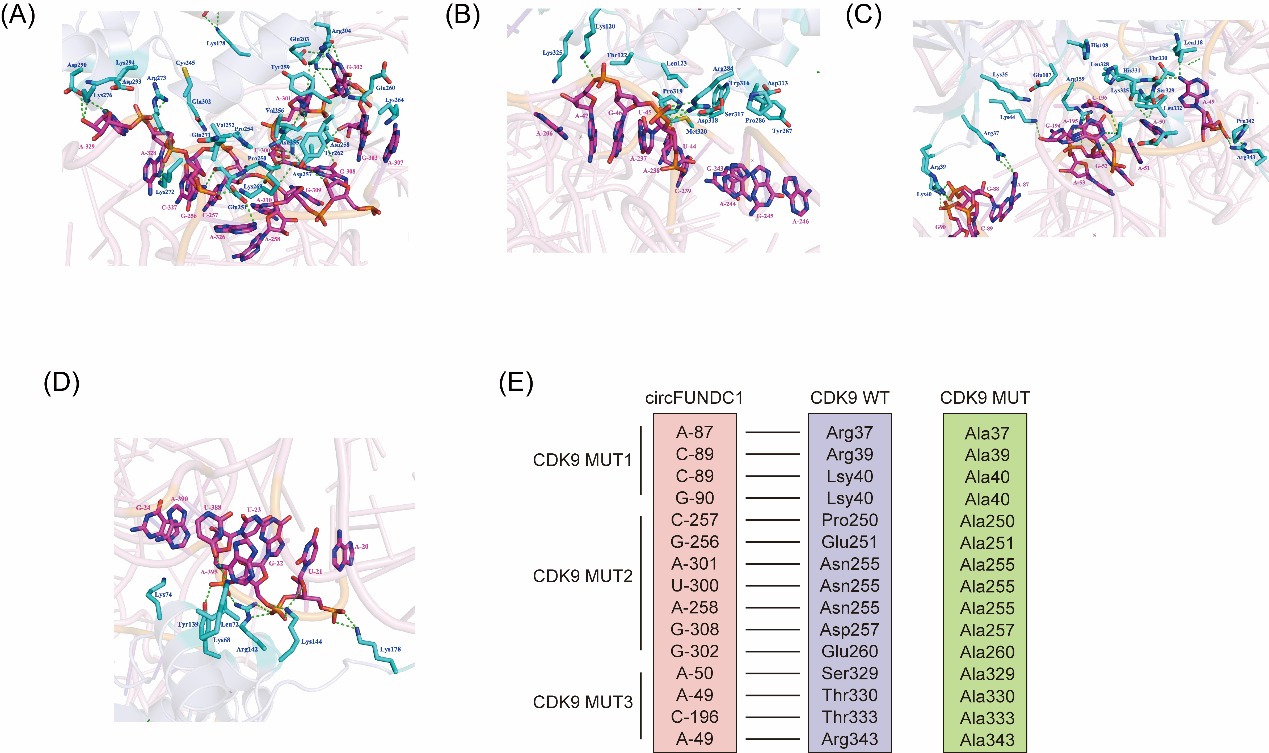


**Figure S2** (**A-D**): Interaction patterns of circFUNDC1 and CDK9, we chose the three most centralized region and mute them. **(E)** Generation of CDK9 mutant 1, 2 and 3 with alanine mutations.

**Figure S3**


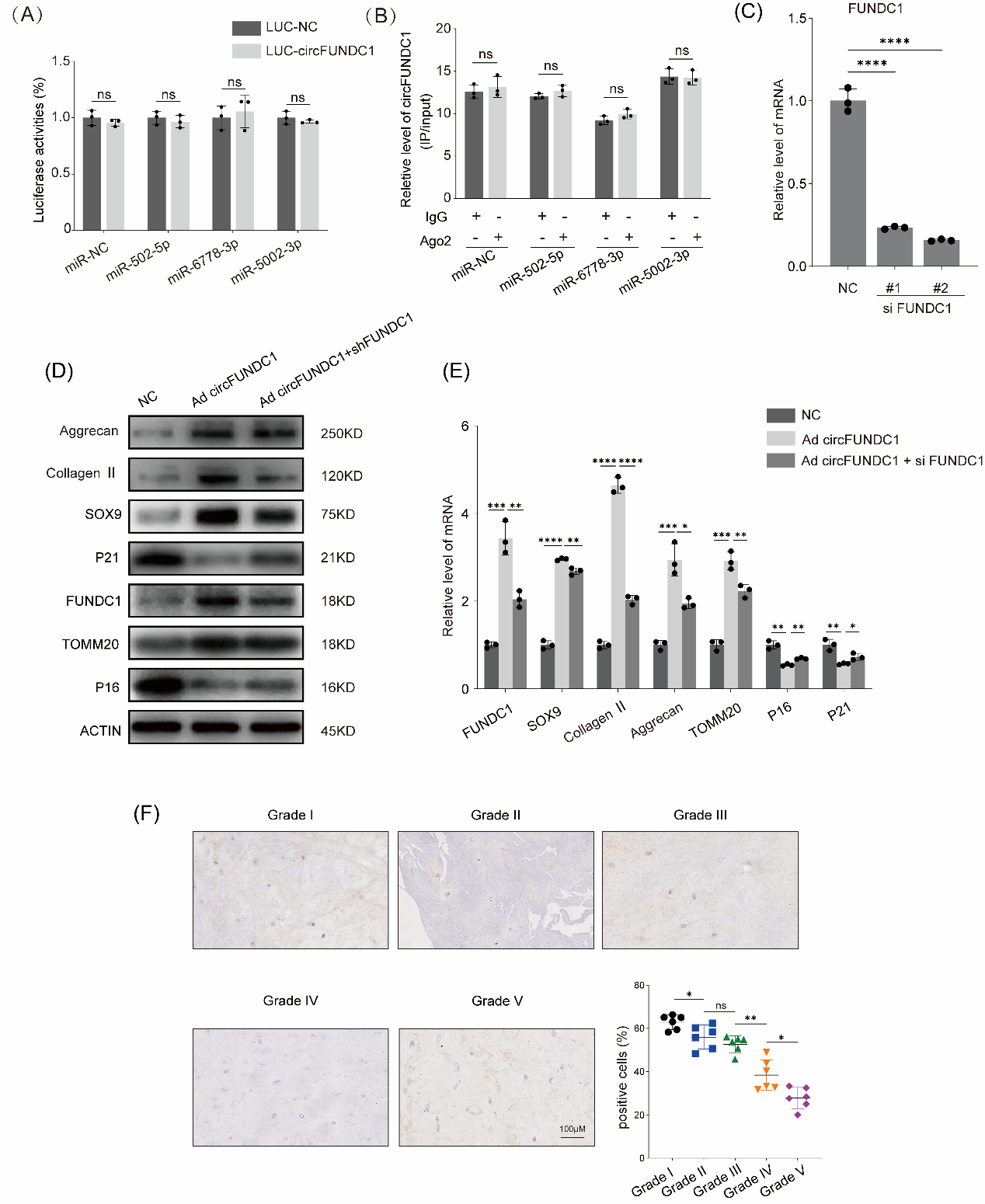


**Figure S3** **(A)** HEK-293 cells were co-transfected with miR-negative control (NC) or three predicted miRNA targets of circFUNDC1, and a luciferase reporter driven by the wild-type or mutated FUNDC1 promoter. **(B)** AGO2 RIP assay was performed to detect the circFUNDC1 levels in NPCs transfected with three predicted microRNAs or negative control. **(C)** Knockdown efficiency of two siRNAs against FUNDC1 was detected by RT-qPCR. **(D&E)** Western blotting and RT-qPCR experiments showed circFUNDC1 could protect NPCs under a deficiency of FUNDC1. **(F)** IHC detection of FUNDC1 in human nucleus pulposus from Grade I to Grade V (scale bar = 100μm). Data are representative images of similar results obtained from three different donors (D and F) or presented as the mean ± SEM from three independent experiments (A, B, C, and E). (ns p>0.05, *p<0.05, **p<0.01, ***p<0.005, ****p < 0.001 vs control or as indicated by the student’s t-test)

**Figure S4**


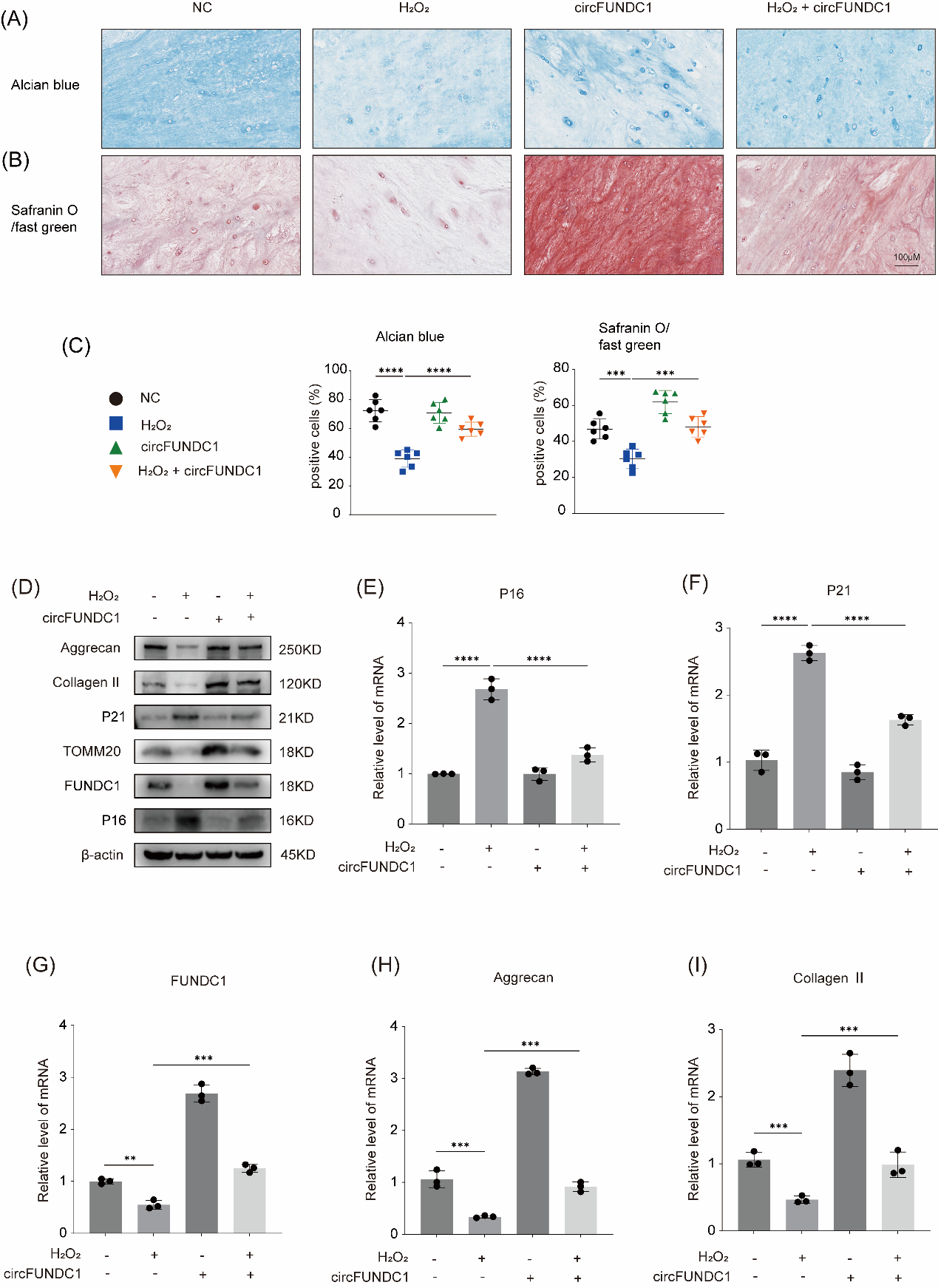


**Figure S4** **(A&B)** Alcian blue and Safranin O/fast green staining of human nucleus pulposus after different treatments. **(C)** Percentage of cells with positive pericellular matrix staining from A and B. Western blotting **(D)** and RT-qPCR analysis **(E-I)** of ex-vivo cultures of nucleus pulposus. Data were representative images of similar results obtained from three different donors (A, B and D) or presented as the mean ± SEM from three independent experiments (E-I). (**p<0.01, ***p<0.005, ****p < 0.001 vs control or as indicated by Student’s t-test)

**Figure S5**

**
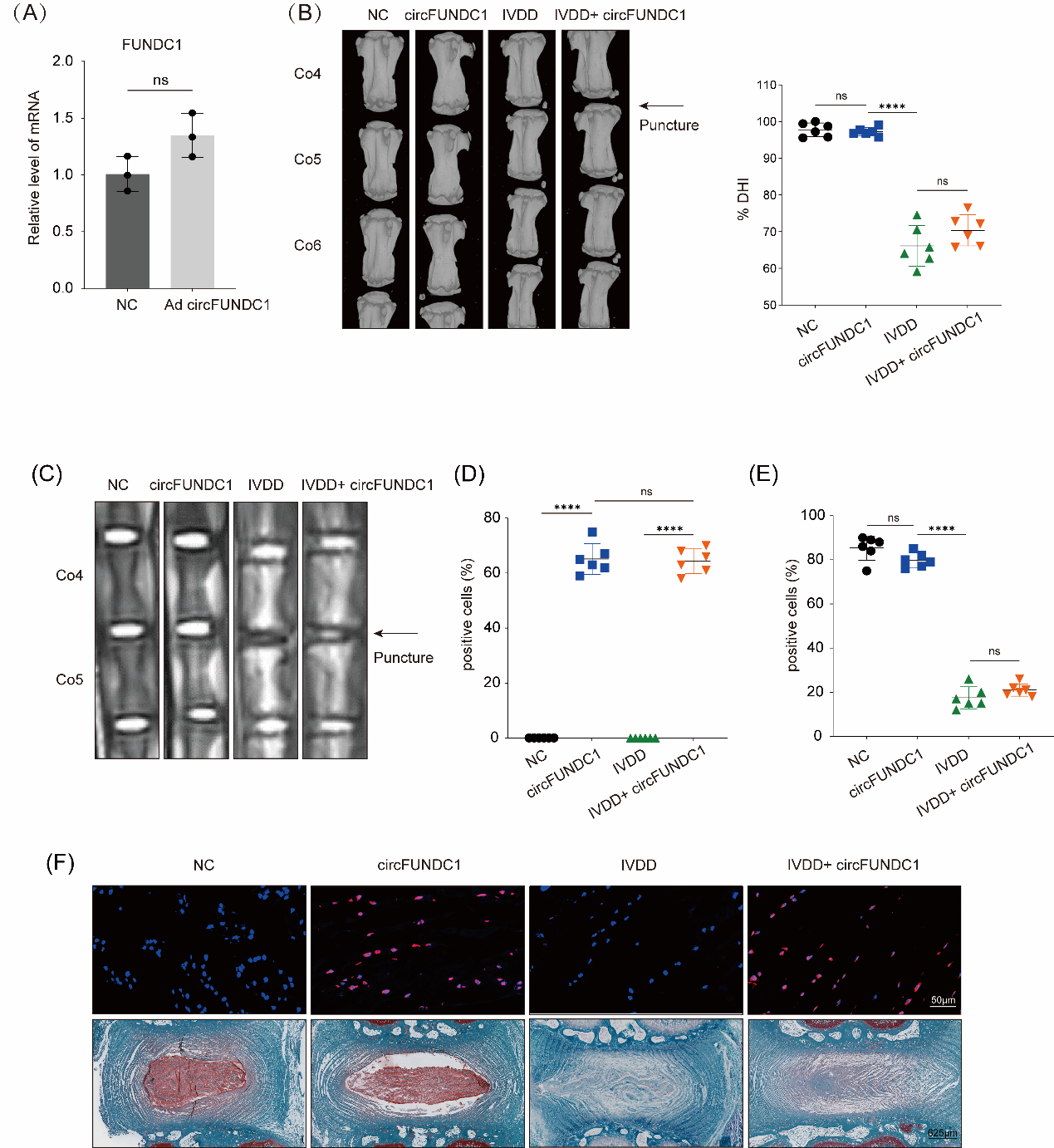
**

**Figure S5** **(A)** qPCR analysis of FUNDC1 expression in rat NPCs infected with adenovirus expressing circFUNDC1 (Ad circFUNDC1) or control virus (NC). **(B)** Micro-CT three-dimensional reconstruction images and disk height index (DHI) of the rat models at 4 weeks after operation. **(C)** Magnetic resonance imaging (MRI) images of the rat model at 4 weeks after operation. **(D&E)** Percent positive cells for each marker and proteoglycan were determined from 3 different fields. **(F)** DAPI staining and a red FISH detection of circFUNDC1 in rat nucleus pulposus infected with Ad circFUNDC1 (scale bar = 50μm) in parallel with Safranin-O/fast green staining (scale bar = 625μm). Data were representative images of similar results obtained from three different donors (B, C and F) or presented as the mean ± SEM from three independent experiments (B, D and E). (ns p>0.05, ****p < 0.001 vs control or as indicated by Student’s t-test).

**Table S1**

| Sequence of circFUNDC1:  ATTGCTAGTCATAGTGGCTATGTGCAGATTGACTGGAAGAGAGTTGAAAAAGATGTAAATAAAGCA  AAAAGACAGATTAAGAAACGAGCGAACAAAGCAGCACCTGAAATCAACAATTTAATTGAAGAAGCA  ACAGAATTTATCAAGCAGAACATTGTGATATCCAGTGGATTTGTGGGAGGCTTTTTGCTCGGACTTGC  ATCTTAAGGACATGAATATTCTCCCATAACGGATTCAACTATGAGAAGAGAAGTGGCAGCAATAAGG  CAGTCTCTCAAAAGTCATACTGCCAGAGTCTCTAGGGCAAGGAGAAACAACTAGCTGGACAATACTC  AATTCACAACTTAGCATTTTGCCATCTGAAGCTTGGCAAACTAGTATCTGCTGTAAAACAACCTATATG |
| --- |
| Fragments of circFUNDC1 |
| Fragment one: 333-54/201-249  TCAATTCACAACTTAGCATTTTGCCATCTGAAGCTTGGCAAACTAGTATCTGCTGTAAAA  CAACCTATATGATTGCTAGTCATAGTGGCTATGTGCAGATTGACTGGAAGAGAGTTGAAAAAGAT/A  TCTTAAGGACATGAATATTCTCCCATAACGGATTCAACTATGAGAAGA  Fragment two: 55-200  GTAAATAAAGCAAAAAGACAGATTAAGAAACGAGCGAACAAAGCAGCACCTGAAATCAACAATTT  AATTGAAGAAGCAACAGAATTTATCAAGCAGAACATTGTGATATCCAGTGGATTTGTGGGAGGCTTTTTG  CTCGGACTTGC  Fragment three:250-332  GAAGTGGCAGCAATAAGGCAGTCTCTCAAAAGTCATACTGCCAGAGTCTCTAGGGCAAGGAGAAACAAC  TAGCTGGACAATAC |

**Table S1** Sequence and three fragments of circFUNDC1

**Table S2**

|  | CDK9 | | RNA | |  | CDK9 | | RNA | |
| --- | --- | --- | --- | --- | --- | --- | --- | --- | --- |
| No. |  |  |  |  | No. |  |  |  |  |
|  | Residue | Atom | Base | Atom |  | Residue | Atom | Base | Atom |
| 1 | Arg37 | NH1 | A-87 | N1 | 18 | Glu251 | O | g-256 | N2 |
| 2 | Arg39 | NH1 | C-89 | O2P | 19 | Asn255 | O | a-301 | N6 |
| 3 | Arg39 | NH2 | C-89 | O2P | 20 | Asn255 | Nd2 | u-300 | O2 |
| 4 | Lys40 | Nz | C-89 | O1p | 21 | Asn255 | Nd2 | a-258 | O1p |
| 5 | Lys40 | Nz | G-90 | O2p | 22 | Asp257 | Od1 | g-308 | O1p |
| 6 | His108 | Nd1 | C-196 | O2p | 23 | Glu260 | N | g-302 | O6 |
| 7 | Heu118 | O | A-49 | N6 | 24 | Lys269 | Nz | c-327 | O1p |
| 8 | Lys120 | Nz | A-47 | O5’ | 25 | Lys272 | Nz | a-328 | O2p |
| 9 | Tyr139 | Oh | U-23 | O1p | 26 | Arg284 | Ne | g-46 | O1p |
| 10 | Arg142 | Ne | G-22 | O2p | 27 | Arg284 | Nh2 | u-45 | O3’ |
| 11 | Arg142 | Nh1 | G-22 | O2p | 28 | Asp290 | Od1 | a-329 | O2’ |
| 12 | Arg159 | Nh1 | A-53 | O1p | 29 | Met320 | N | u-44 | O4 |
| 13 | Lys178 | Nz | U-21 | O2p | 30 | Ser329 | O | a-50 | O2’ |
| 14 | Gly202 | O | G-302 | N1 | 31 | Thr330 | O | a-49 | N6 |
| 15 | Gly202 | O | G-302 | N2 | 32 | Thr333 | Og1 | c-196 | N4 |
| 16 | Arg204 | Nh1 | G-302 | N3 | 33 | Arg343 | Nh1 | a-49 | O1p |
| 17 | Pro250 | O | c-257 | O2’ | 34 | Arg343 | Nh2 | a-49 | O1p |

**Table S2** Predicted hydrogen bonding in circFUNDC1 and CDK9.

**Table S3**

| Primers, antibodies and sequences used in this study | | | | |
| --- | --- | --- | --- | --- |
| Gene | Forward primer: | | Reverse primer | |
| ADAMTS5 | GGGCACTGGCTACTATGTGG | | CGTCACAGCCAGTTCTCACA | |
| Aggrecan | CTACCAGTGGATCGGCCTGAA | | CGTGCCAGATCATCACCACA | |
| GAPDH | GGAGCGAGATCCCTCCAAAAT | | GGCTGTTGTCATACTTCTCATGG | |
| FUNDC1 | CCTCCCCAAGACTATGAAAGTGA | | AAACACTCGATTCCACCACTG | |
| SOX9 | GCTCTGGAGACTTCTGAACGA | | CCGTTCTTCACCGACTTCCT | |
| U6 | CTCGCTTCGGCAGCACA | | AACGCTTCACGAATTTGCGT | |
| β-actin | AGAGCTACGAGCTGCCTGAC | | AGCACTGTGTTGGCGTACAG | |
| linear FUNDC1-206bp | GGTATCATGGCGACCCGGAA | | AGAAATCCTGCACACCAGCCA | |
| linear FUNDC1-288bp | CGTTACTGGCTGGTGTGCAG | | GCAAGTCCGAGCAAAAAGCC | |
| circFUNDC1-256bp | AGAAGTGGCAGCAATAAGGCA | | GCTGCTTTGTTCGCTCGTT | |
| MMP3 | CCTACAAGGAGGCAGGCAAG | | CCCGTCACCTCCAATCCAAG | |
| MMP13 | TCGGCCACTCCTTAGGTCTT | | AAGTGGCTTTTGCCGGTGTA | |
| COL2 | ATGACAATCTGGCTCCCAAC | | GAACCTGCTATTGCCCTC | |
| AD4 | GAGGAGGAGATCGTGTTTCCA | | CCAGCTCTAGTAGCAGCGTC | |
| circEIF3J_Div | AAAAGAGAAAGAACGGCAACA | | CCTGGTTTTACTTCTGCTTCC | |
| circPAIP2_Div | AGCTCGAGATCTCCCACAAA | | ACTGCTGCGACTTGGATCTT | |
| circRNA2_Div | TGTGGGAAGCAAGGAGGAACTTGA | | TCTTGAAGTGAGCTGTGTCAGGCA | |
| circRSRC1 | TATGGCTCCAGAAGGAAACG | | GGACCGTCTACGGTGTTTCT | |
| circMIER1 | GATTATGGTGGGCTCCATGT | | TTCCTCTTCTTCCTCTTCCTCA | |
| circSSR1 | TTGTGCCACTGCTAACAAGC | | GGAAGAAGATGTGTCTGGTGAA | |
| circWDR60-F | GAAAGATGAAGACTCTGAAAGAGG | | CTGAGGTCATCTGCTTTCCA | |
| circMAN1A2-1 | ATGGGCAAAGATGGATTGAA | | TGCTCGAATTTCCTCTCTTGA | |
| circRBM33 | CCACGAAGCTGAGTTGACAG | | GCCAGATAGCAAATCTTCTCCA | |
| circMAN1A2-2 | GGAGGCCTACTTGCAGCATA | | GCTTCTTCCAAGGCCTTCTC | |
| circMAN1A2-3 | GCAATTGGCTGAGAAACTCC | | GCTTCTTCCAAGGCCTTCTC | |
| circNAP1L4 | TCCACGGAATCTGAAGGAAC | | GGAGAAGACGAGGATGATGC | |
| circBPTF | TCCAAGTGACTCCCCATTTT | | CTTCTGCTGGACCCACACTT | |
| circCLTC | TGCCCTATTTCATCCAGGTC | | GGGCCTGCTCTCCTACTTTT | |
| circCDK11B | TGGTTTGATGGCCAAAGAAT | | CCGCATGGAGATCACAATAA | |
| Mimics | | sense（5'-3'） | | antisense（5'-3'） |
| miR-5002-3p | | UGACUGCCUCACUGACCACUU | | GUGGUCAGUGAGGCAGUCAUU |
| miR -502-5p | | AUCCUUGCUAUCUGGGUGCUA | | GCACCCAGAUAGCAAGGAUUU |
| miR-6778-3p | | UGCCUCCCUGACAUUCCACAG | | GUGGAAUGUCAGGGAGGCAUU |
| ASO, SiRNAs and shRNA | | | | |
| ASO-h-hsa_circ_0007290_001 | Target sequence: CCTATATGATTGCTAGTCA From RiboBio | | | |
| ASO-h-hsa_circ_0007290_002 | Target sequence: ACAACCTATATGATTGCTA From RiboBio | | | |
| SiRNA and shRNA for FUNDC1 | Constructed by RiboBio , due to company policies, the sequences will not be disclosed. | | | |
| Antibodies | | | | |
| β-actin | Cell Signalling Technology, Cat.4967S | | | |
| FUNDC1 | Abcam, Cat.ab224722 | | | |
| P21 | Abcam, Cat.ab109520 | | | |
| P16 | Abcam, Cat.ab151303 | | | |
| collagen II | Abcam, Cat.ab307674 | | | |
| Sox9 | Abcam, Cat.ab185966 | | | |
| CDK9 | Proteintech, Cat. 11705-1-AP | | | |
| TOM20 | Proteintech, Cat. 11802-1-AP | | | |
| Promoter area sequence of human FUNDC1: -2000 to -1  ATGCAATTTATGCCAGAATTCCTTGTTTTTCCATTAAAATGAATACTGAGAGGACTTTTGTTTAATTG  TACAGCAGATGCGGGTGCAACGCAAGTTCAAGACAAGTTTCTGGGTTGGTTTATGTGGACTTGGGTTA  GCTGCTTGGTGATCAATATGGCTTTGTATTCATAGAATCCCATGGGGTTTCTGGGTGAAGACCTTGACC  TTGTTCCTCTGTCAGATTTAAATAATAAGAATGATGCTGGCCGGGTACGGTGGCTCACCCCTGTGATCC  CAGCACTTTGGGAGGCCCAGGTGGGTGGATCACTGAGATCAGGAGTTCGAGACCAGCCTGGGCAACAT  GGTGAAACCCCCTCTCTACTAAAAATACAAAAATTAGCCGGGCGTGGTGGCACGTGCCTGTAATCCCA  GCTCCTTGGGATGTTGGGAGGCTGAGGCAGGAGAATCACTTGAATTCGGGAGGTGGAGGTTGCAGTGA  GCCGAGATTGTGCCATTGCACTCCAGCCTGGGCAACAGAGCGAGATTCCATCTCAAACAAACAAACAA  AAGAATGACACTAAAAAATTATGTCCTCTTGCCAGGTCAGGAATTACGAGGTTTCTAGGCCTTTCTTTA  CCTCCTTTGGATTCCTGGCAGGAACTCAAGAGAGAGAGCAGGTAAACAGATTCCAACTTCTAGCAGCAG  GGCATTAGAAAACTTGTGGTCCCTTTGTGAGCGCTTGCGCCCTTAGAAAAGTATGGCTTGAAACAGGGT  TTCTCTTATCTTTTACAAGCCTTTTTCCTCAAGCATTTGAATTCTGGTAATGTTCAAAATACTGTTAAAAA  ATTATTCTGACAGTATGCAAGTGATGGGCCTGACAAAAAAATTATGGGACACTTCTTAAAATGGTAAGG  AAGACTTTGTTCATGACTACTGCAATAGGTATCACAACTATTGCAATACAGCCTGGCATGGTGGCTCAC  ATCTGTAATCCCAGCACTTTGGGAGGCAGAGGCAGGAAGACTGCTTGAGCCCAGAAGTTTAAGAGCAGC  CTGGGCAACACAGGGAGACCTACTCTCCACAAAAAATAAAAATAAATTAGCCGGGAGTGGTGATGCAC  GCCTGGAGTCCTGGGTACTCTGGAGGCTGAGGTGGGCCGATCTCTTGGGCCCAGGAGTTTGAGGCTGCA  GTGAGCCATGATAGCGCCACTGCACTCCAGCCTGGTGACAGAGCAATACCTTTTCTCATAAAACAAAGA  CTATGGCAATAGGGGTGAGTGATGGGTCTCAACTCTGAATACAGCAGGGAATTTATAGTAAATGATCAG  AGTGAATGGATCAATGGGTGGAAGATTACCAAGAGTAGACATGAAGGGTAGGGGTATTCTTGCTAAAGC  CAATTTAACAGGATTTTTGCTGGTGGCAGAGTGATCAGATATCAAGAGTGGGGTTGAGGAATTCGATCGG  ATATTGAGGGTGATTCGATATTAAGGGTGGGGGAGTTCTTACTAAACTGACTTAACTGCATTCTTACCAC  AAATGGACTAGGCAGGTCAAAGACGACCCAAGGAGGAGACCCAGTCAAAAACAGCTCAGAGGAGCCTC  CTGACTAAAGTTTGGTCAAGGAGAGAGTCTTTGTCAATACCCAGCTGGTTCTCCCTTTGTACCACCATGTG  GCGCCCTTCACCCCATTCCTTCAGCCCTTAAGGCTCTTTTAAAACAAAATAATCAAATTCAAGTTACGGAA  ATTCTAGGAGGGGCGGTCCCTCCCCCTTCCTAGTTGCGGAAGGCAACGGACTACAAGTCCCCTACGCTCT  CGCGGGGCTATGATCCGCCCGGCCCTCCCCACATTCCGGAGTTTCGCGCCTGCGCAGAAGGCAGAAGGTC  CTGCGCTGAGAGAAGGGGGCGGAGACAGGACTACGCGCCTGGAGTAGGAGAAGGAGGAAAAAAGAGAC  CATAGACTTCCATCCTGGCCTAGAGCGGCCCTTAAAGTGCCAGGGAGAGGAGGGCGGGTGGGGACCA | | | | |
| Mutated Promoter area sequence of human FUNDC1:  ATGCAATTTATGCCAGAATTCCTTGTTTTTCCATTAAAATGAATACTGAGAGGACTTTTGTTTAATTGTAC  AGCAGATGCGGGTGCAACGCAAGTTCAAGACAAGTTTCTGGGTTGGTTTATGTGGACTTGGGTTAGCTGC  TTGGTGATCAATATGGCTTTGTATTCATAGAATCCCATGGGGTTTCTGGGTGAAGACCTTGACCTTGTTCC  TCTGTCAGATTTAAATAATAAGAATGATGCTGGCCGGGTACGGTGGCTCACCCCTGTGATCCCAGCACTTT  GGGAGGCCCAGGTGGGTGGATCACTGAGATCAGGAGTTCGAGACCAGCCTGGGCAACATGGTGAAACCC  CCTCTCTACTAAAAATACAAAAATTAGCCGGGCGTGGTGGCACGTGCCTGTAATCCCAGCTCCTTGGGATG  TTGGGAGGCTGAGGCAGGAGAATCACTTGAATTCGGGAGGTGGAGGTTGCAGTGAGCCGAGATTGTGCCA  TTGCACTCCAGCCTGGGCAACAGAGCGAGATTCCATCTCAAACAAACAAACAAAAGAATGACACTAAAAA  ATTATGTCCTCTTGCCAGGTCAGGAATTACGAGGTTTCTAGGCCTTTCTTTACCTCCTTTGGATTCCTGGCA  GGAACTCAAGAGAGAGAGCAGGTAAACAGATTCCAACTTCTAGCAGCAGGGCATTAGAAAACTTGTGGTC  CCTTTGTGAGCGCTTGCGCCCTTAGAAAAGTATGGCTTGAAACAGGGTTTCTCTTATCTTTTACAAGCCTTT  TTCCTCAAGCATTTGAATTCTGGTAATGTTCAAAATACTGTTAAAAAATTATTCTGACAGTATGCAAGTGAT  GGGCCTGACAAAAAAATTATGGGACACTTCTTAAAATGGTAAGGAAGACTTTGTTCATGACTACTGCAATA  GGTATCACAACTATTGCAATACAGCCTGGCATGGTGGCTCACATCTGTAATCCCAGCACTTTGGGAGGCAG  AGGCAGGAAGACTGCTTGAGCCCAGAAGTTTAAGAGCAGCCTGGGCAACACAGGGAGACCTACTCTCCAC  AAAAAATAAAAATAAATTAGCCGGGAGTGGTGATGCACGCCTGGAGTCCTGGGTACTCTGGAGGCTGAGG  TGGGCCGATCTCTTGGGCCCAGGAGTTTGAGGCTGCAGTGAGCCATGATAGCGCCACTGCACTCCAGCCTG  GTGACAGAGCAATACCTTTTCTCATAAAACAAAGACTATGGCAATAGGGGTGAGTGATGGGTCTCAACTCT  GAATACAGCAGGGAATTTATAGTAAATGATCAGAGTGAATGGATCAATGGGTGGAAGATTACCAAGAGTA  GACATGAAGGGTAGGGGTATTCTTGCTAAAGCCAATTTAACAGGATTTTTGCTGGTGGCAGAGTGATCAG  ATATCAAGAGTGGGGTTGAGGAATTCGATCGGATATTGAGGGTGATTCGATATTAAGGGTGGGGGAGTTC  TTACTAAACTGACTTAACTGCATTCTTACCACAAATGGACTAGGCAGGTCAAAGACGACCCAAGGAGGAG  ACGAACTACTAAACAGCTCAGAGGAGCCTCCTGACTAAAGTTTGGTCAAGGAGAGAGTCTTTGTCAATAC  CCAGCTGGTTCTCCCTTTGTACCACCATGTGGCGCCCTTCACCCCATTCCTTCAGCCCTTAAGGCTCTTTTA  AAACAAAATAATCAAATTCAAGTTACGGAAATTCTAGGAGGGGCGGTCCCTCCCCCTTCCTAGTTGCGGA  AGGCAACGGACTACAAGTCCCCTACGCTCTCGCGGGGCTATGATCCGCCCGGCCCTCCCCACATTCCGGAG  TTTCGCGCCTGCGCAGAAGGCAGAAGGTCCTGCGCTGAGAGAAGGGGGCGGAGACAGGACTACGCGCCT  GGAGTAGGAGAAGGAGGAAAAAAGAGACCATAGACTTCCATCCTGGCCTAGAGCGGCCCTTAAAGTGCC  AGGGAGAGGAGGGCGGGTGGGGACCA | | | | |
| Promoter area sequence of rat FUNDC1: -2000 to -1  GACATTCAAATAAAAATCCATATTTACTTTTGCATTTTCTGAGTTTGGATTGATTGGCAATCTCCAACCAG  ATTTCATTTTAAAAGAATGTAAGAATAGAATGGAAACTGCATTCATCACAAGACTGTGAAAATCTCATG  ATGCACATTTTGAGCTATAGGCATGCTGTAACTATGTAATTGTTCTGGATTTTCCATAAGCCTGAGATTAA  CACAAGCTCACACATCCAGGCAGCAGATGTTGGTGTGCCTAGAGGCTCCAGATGCTGGATATTTCCATTCT  ATTTACACATTCTATTTATGTATATACTCTGAAGATAGTGGAATCCCATTAGACCATAGCAAAGAAAATTT  GGTTTTCTAAACAACTGAAAAGGAAAGTAGAATTTAAGCAAACAGACTAAGAATTATTTGCATTGATAAG  GCAAGAACTAAGAAATACCTGTTATTTAATAGTTATTATAATTAGTATAATAAGATTCATGATAATGAAG  GTAAGCAATCCAATTTGCAGGCATATTACCTGCTTAGAGGTATTCTGAAATGACTGAAAGCCATTTTCAAG  CTTATATAAATGAAAAATTAATAAGCAAGAATGGTTTTTAAACTAGCAAGATTTGAAATATCAAGTGCAA  AGCTATTATTTTTAAATAATATTAATACTGAGCAGGCTGTTCATTTTAACCATCCAGCTAATTCAGATAGA  GTACATATTTGAAGTAAGTTTGGTGCTAATTTACGTGAACTTGGATTAGCTATTTGGTATTCAACATGTTAT  GTTCTACAGAATCCATGGAACTTTGTGTTTAAAAAACTTGACTTTATCCCTATGTCACATTTAAATAATAAC  AATTCTTAAAATCACAGCTTGGTGGTGAATGTTTGTAATCACAGTACTGGAGAGAGAGACAGAGGAATCC  AGCCAGGATCAGAGCCCAGCCTGGGCTACACAGCGAGGCCATGTGTAAAATCAAACACAGCAACAGAGT  GATACATGCCTTAATCCCAATACTGGAGAAGGAGGTGGATCTGTGCAAGTTTGAGGCCAGAATGTTCTAC  ATAGCAAGTTCCAGAGAATCCTGGATTATGTGAGGTGTCTAAAAATAAACAAACAAACAAAAATACAAG  CTAAAGTCTGCTCAACTAACAGGGCACTAAGGAAAAAAAGCTCGTCTCATGGAGAACTTTTGGAAGTCTG  AGAAAATTGTGGCTTGAAACAGCGTGTTCTCTTTTGTTTCTATAGTGTTTAAAACAGGTTTTTGTTTTTTGTT  GTTTTACAAAACATCTTTAAAATATTTTCCCTGACACTTGTTAAAATAGATAATGGGTTTATTTTAATTTGG  AATACAGACAAAAAGCTGGAGATTTATAGTGTATAATCTGAGGGTGTCAAGGTGTAGAATCCTACCAAGA  ATCAGGGCATGGGGAGTTTATTGCTAAGGTAATTTAATAGCGTTCTTAACTGGAGAAAGTCTGGGTTATTA  GCCATCAAGAGAGGGGCAAGGCTGAAATTTCTGTTAAAGTCAGCAGGATTCTTGCAAGAACTACCCTTGA  TCTTAGCTAAAAGGCCGAGAAGTGATCCTTGCAAGAACTAAATGTTAAATATAGGGCCAAAAAAAAAAG  AACGCCTAATCAAAAAAAAAAAAAAAAAAAAAAAAAAAAAAAAAAAAAAAAACAACTTCAGAAGAGCA  TCCTGAATAAAGACTTGTTAAGAACAGTCTGCCAACACTAAACTGGTTATTCTCTTCTATTACCCTGTGCCT  CCATTCCCTCCATTCCTTTAGCTTAAAAAAAAAAATCTTGGAAACTCTAGAGCGCGTTGGGGGAATGCCCA  GAGTACAGTGGAAAAGAGCACGAACTACATTTCCCATAAGTAAACACGGGGCGGGGACTCAGCTATTCCT  CCCCAAATCCCAAGCCTTGCGTCTGCGCACAAAGTGTTAAAAGGGCTGTGCAGGGAAGCGTGGGCGGTGA  CGAGCCTGTGTGTGAGAGTTTGTGTGT | | | | |
