# Supplementary material for: CircFUNDC1 interacts with CDK9 to promote mitophagy in nucleus pulposus cells under oxidative stress and ameliorates intervertebral disc degeneration
Source: Cell Death Dis. 2025 Feb 13;16(1):94. doi: 10.1038/s41419-025-07425-2 (PMC11825710; doi:10.1038/s41419-025-07425-2)

Experiments are independently performed three times, and representative images are shown

Figure 2

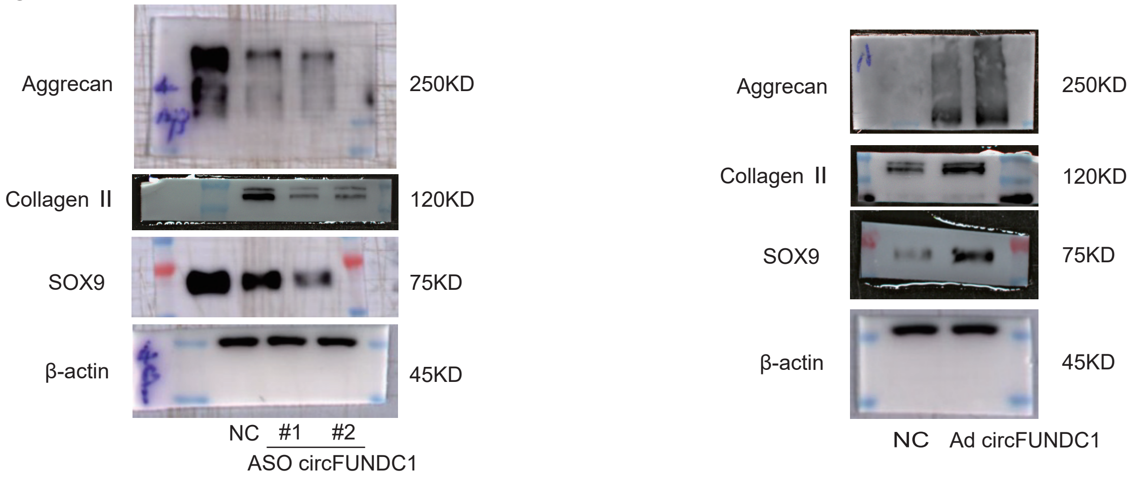

Figure 3

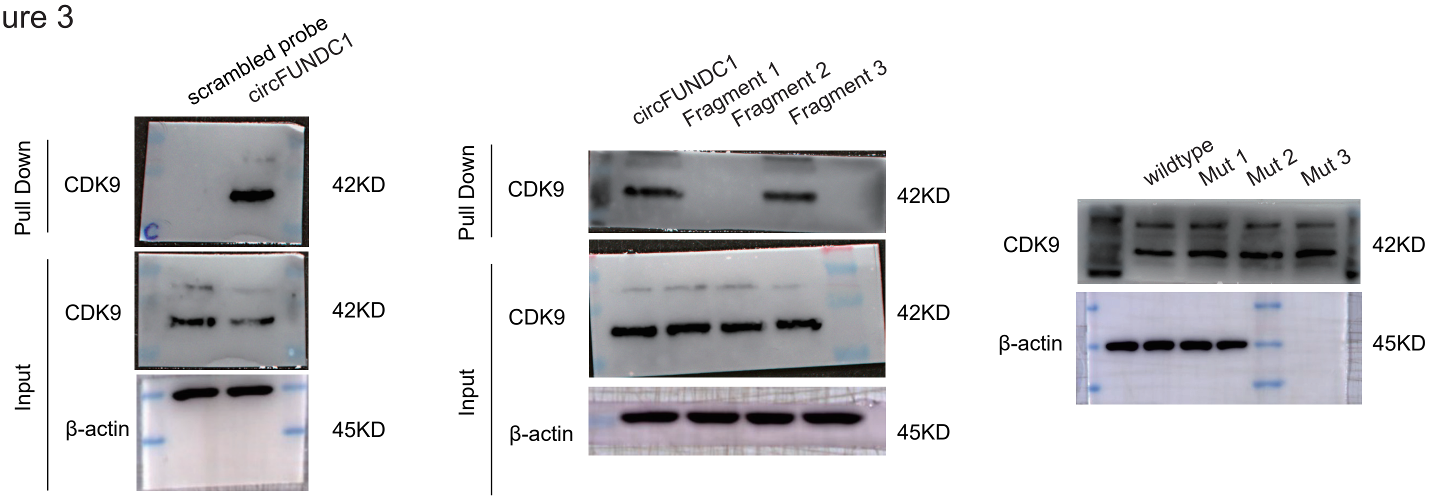

Figure 4

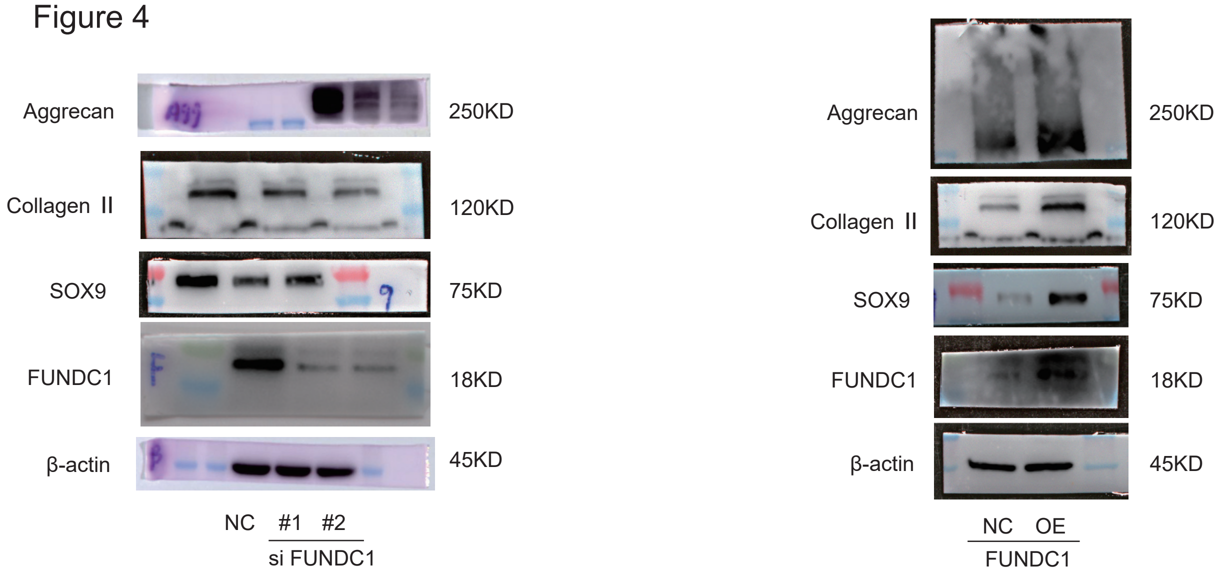

Figure 5

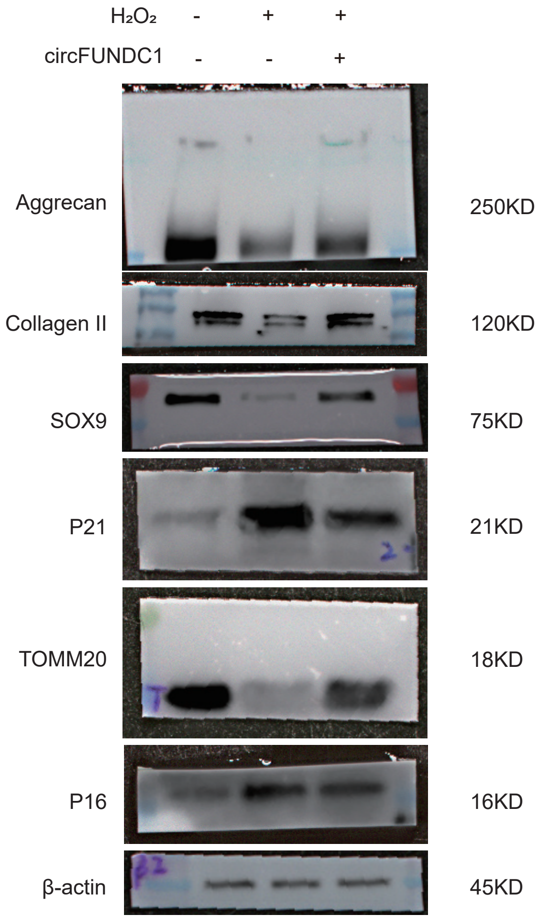

Supporting information

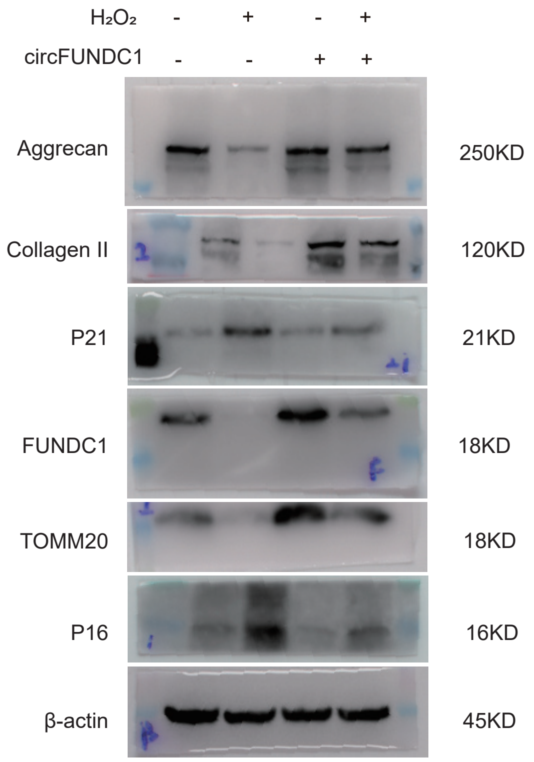

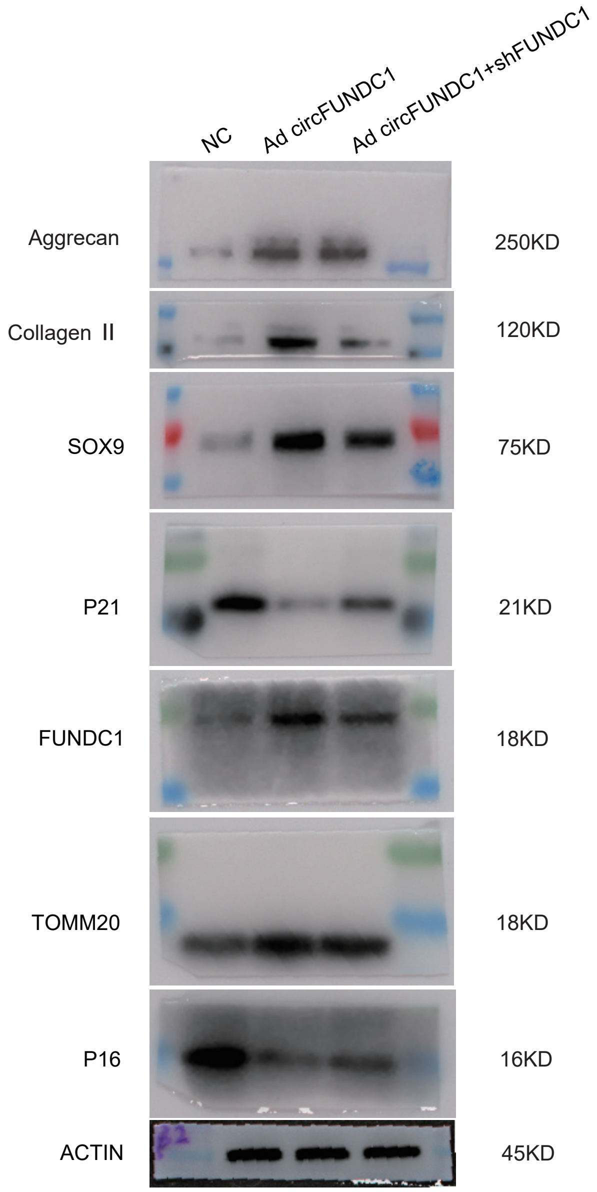

Supplement: Supplementary file 2 — Supplemental Material-WB [file 41419_2025_7425_MOESM2_ESM.pdf]
